# M-Phase Phosphoprotein 9 regulates ciliogenesis by modulating CP110-CEP97 complex localization at the mother centriole

**DOI:** 10.1038/s41467-018-06990-9

**Published:** 2018-10-30

**Authors:** Ning Huang, Donghui Zhang, Fangyuan Li, Peiyuan Chai, Song Wang, Junlin Teng, Jianguo Chen

**Affiliations:** 1 Key Laboratory of Cell Proliferation and Differentiation of the Ministry of Education and State Key Laboratory of Membrane Biology, College of Life Sciences, Peking University, 100871 Beijing, China; 2 Center for Quantitative Biology, Peking University, 100871 Beijing, China

## Abstract

The primary cilium is elongated from the mother centriole and has diverse signaling roles during development and disease. The CP110-CEP97 complex functions as a negative regulator of ciliogenesis, although the mechanisms regulating its mother centriole localization are poorly understood. Here we show that M-Phase Phosphoprotein 9 (MPP9) is recruited by Kinesin Family Member 24 (KIF24) to the distal end of mother centriole where it forms a ring-like structure and recruits CP110-CEP97 by directly binding CEP97. Loss of MPP9 causes abnormal primary cilia formation in growing cells and mouse kidneys. After phosphorylation by Tau Tubulin Kinase 2 (TTBK2) at the beginning of ciliogenesis, MPP9 is targeted for degradation via the ubiquitin-proteasome system, which facilitates the removal of CP110 and CEP97 from the distal end of the mother centriole. Thus, MPP9 acts as a regulator of ciliogenesis by regulating the localization of CP110-CEP97 at the mother centriole.

## Abstract (web summary)

Ciliogenesis is negatively regulated by the CP110-CEP97 complex, although the mechanism controlling mother centriole localization is poorly understood. Here, Huang et al. show that KIF24 recruits MMP9 to the mother centriole, where it regulates ciliogenesis by controlling CP110-CEP97 recruitment.

## Introduction

Centrosomes are the major microtubule-organizing centers in animal cells, and one centrosome contains mother and daughter centrioles that are distinguished by the distal and subdistal appendages present on the mother centriole^1,2^. When cells exit from the cell cycle, the mother centriole can convert into the basal body. The primary cilium, a membrane-bound, hair-like organelle, can then elongate from the basal body in most quiescent vertebrate cells. Primary cilia sense mechanical and chemical signals from the extracellular milieu and transduce them into the nucleus, which is necessary for embryonic development and maintenance of homeostasis^3–5^. Defects in the formation and function of primary cilia cause severe diseases (ciliopathies), such as Bardet-Biedel syndrome (BBS), Joubert syndrome, Meckel-Gruber syndrome (MKS), and nephronophthisis (NPHP)^6,7^.

Since the primary cilia are physiologically important, ciliogenesis is tightly controlled in a temporally and spatially specific manner. So far, many positive regulators of ciliogenesis, such as components of the distal appendages and transition zone as well as intraflagellar transport (IFT), have been reported to function during the different stages of this process^8–10^. However, negative regulators of ciliogenesis are largely unknown. CP110 and its interacting protein CEP97 are localized at distal centrioles and are the first proteins identified to negatively regulate the early steps of ciliogenesis. Loss of either CP110 or CEP97 causes premature cilia formation or abnormal centriole elongation in proliferating cells, while their overexpression can repress cilia formation upon serum starvation^11^. CEP97 mainly cooperates with CP110 and stabilizes the localization of CP110 at the distal ends of centrioles^11^, while the precise function of CEP97 is less studied and remains to be validated. In addition to its interaction with CEP97, CP110 also cooperates with a series of proteins pivotal for ciliogenesis, including KIF24^12^, CEP104^13^, and CEP290^14^.

Although the essential roles of CP110 and its cofactor CEP97 in suppressing ciliogenesis have been revealed, the regulatory mechanisms underlying the mother centriole localization of CP110 and CEP97 in cycling cells and quiescent cells are poorly understood. KIF24, a member of the kinesin-13 family of proteins, interacts with CP110 and negatively regulates ciliogenesis in two different ways: by controlling ciliary axoneme elongation through the depolymerization of centriolar microtubules and by recruiting the CP110-CEP97 complex to the distal end of the mother centriole^12^. Tau Tubulin Kinase 2 (TTBK2), a microtubule plus-end tracking kinase, was recently shown to be recruited to the distal appendages by CEP164, CEP350, and FOP, and to function in the maturation of the basal body at the initial step of ciliogenesis^15,16^. Accumulation of TTBK2 at the basal body coincides with the loss of CP110 from the basal body at the beginning of ciliogenesis, and loss of TTBK2 perturbs the displacement of CP110 from the distal end of the mother centriole and inhibits ciliogenesis^17^. However, precisely how TTBK2 modulates the localization of CP110 and promotes ciliogenesis is still unknown.

M-Phase Phosphoprotein 9 (MPP9) was first identified as a protein phosphorylated during mitosis^18^. Subsequently, MPP9 was shown to be a centrosome component and to localize to both the distal and proximal ends of two centrioles^19,20^. Interestingly, akin to CEP97 and CP110, the localization of MPP9 at the distal end of the mother centriole disappears when ciliation begins, but the mechanism underlying this phenomenon is not clear^20^. In this study, we show that MPP9 is localized at the distal ends of centrioles in a small ring-like structure and recruits the CP110-CEP97 complex at the distal end of the mother centriole in ciliary cells. At the beginning of cilia formation, MPP9 undergoes TTBK2-mediated phosphorylation and degradation via the ubiquitin-proteasome system (UPS) and removes itself and the CP110-CEP97 complex from the distal end of the mother centriole, which subsequently promotes cilia formation. Together, our results uncover a role for MPP9 as a switch for ciliogenesis, which functions by regulating the CP110-CEP97 complex at the mother centrioles/basal bodies.

## Results

### MPP9 forms a ring-like structure at the centriole distal-end

**Fig. 1:**
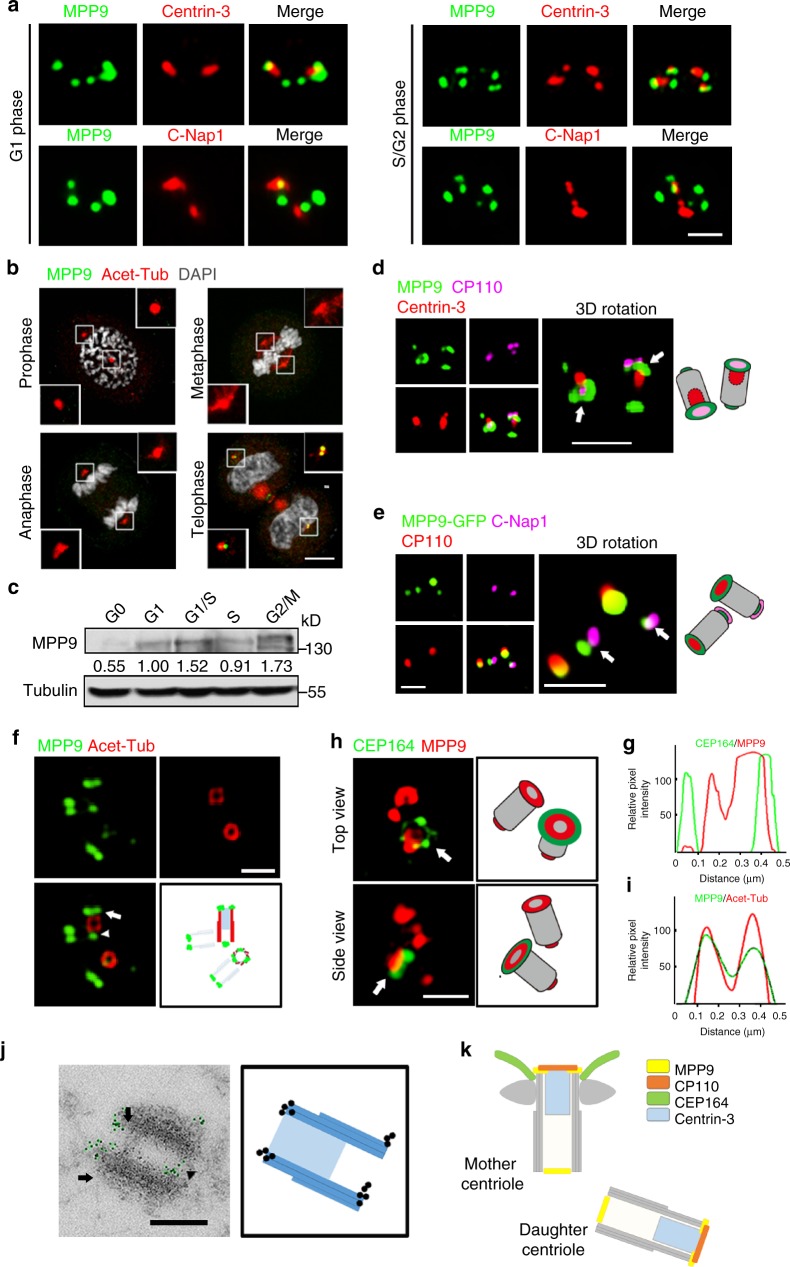
MPP9 represents the ring-like structures at the distal ends of the centrioles. **a** Immunofluorescence of MPP9 (green) and Centrin-3 (red, upper) or C-Nap1 (red, lower) in U2OS cells. **b** Immunofluorescence of MPP9 (green) and acetylated-tubulin (Acet-Tub, red) in mitotic HeLa cells. DNA was stained with DAPI (white). **c** Immunoblots of MPP9 during the cell cycle in hTERT RPE-1 cells. Tubulin was used as a loading control. Relative amounts of MPP9 were quantified and normalized to tubulin. **d** 3D-SIM images of U2OS cells triple-immunostained with antibodies against MPP9 (green), CP110 (purple), and Centrin-3 (red). Arrows show distal ends of the centrioles. **e** 3D-SIM images of MPP9-GFP (green) overexpressing U2OS cells co-immunostained with antibodies against CP110 (red) and C-Nap1 (purple). Arrows show proximal ends of the centrioles. **f**–**i** 3D-SIM images of U2OS cells co-immunostained with antibodies against MPP9 (**f**, green) and Acet-Tub (**f**, red) or MPP9 (**h**, red) and CEP164 (**h**, green). **g**, **i** are the intensity plots of the rings from **f**, **h**, respectively. **j** Immuno-electron microscopy images. U2OS cells were labeled with anti-MPP9 antibodies followed by anti-rabbit IgG-gold (10 nm) secondary antibodies. Green, gold particles. Schematics of immuno-electron microscopy images are shown. **k** Schematic of MPP9 localization at centrosomes. Scale bars: 5 μm (**b**); 1 μm (**a**); 500 nm (**d**, **e**)

A previous study has shown that MPP9 is localized at both the distal and proximal ends of the mother and daughter centrioles^20^. To confirm the localization of MPP9 at the centrosomes, we first generated anti-human MPP9 antibodies (Supplementary Fig. 1a, b). Immunostaining results showed that MPP9 was localized to the centrosomes as 4-dots in U2OS cells (Fig. 1a), consistent with the former study^20^, and the localization of both human and mouse MPP9 ectopically expressed in U2OS cells displayed the same pattern (Supplementary Fig. 1c). Subsequently, we co-stained U2OS cells with both MPP9 and either Centrin-3 or C-Nap1, markers of the distal and proximal ends of the centrioles, respectively. The two larger dots representing MPP9 were colocalized with Centrin-3, and the other two smaller dots were localized close to C-Nap1 in G1 phase cells (Fig. 1a, left panel). Following procentriole formation and elongation during the S/G2 phase, MPP9 was also localized at the distal ends of the procentrioles, forming a pattern consisting of 6-dots (Fig. 1a, right panel). To delineate the domains of MPP9 that serve to maintain its centrosome localization, we constructed a series of MPP9 truncation mutants. It was demonstrated that the middle region of MPP9, including the coiled-coil domain (496–652 aa), was required for its centrosome localization (Supplementary Fig. 1d, e). Interestingly, the localization of MPP9 at the centrosomes was markedly decreased after mitotic entry and then gradually recovered when the cells entered telophase (Fig. 1b and Supplementary Fig. 1f).

We also examined MPP9 protein level in synchronized human telomerase reverse transcriptase immortalized retinal pigment epithelial (hTERT RPE-1) cells. MPP9 was mostly absent in the G0 phase (Fig. 1c). Although the centrosome localization of MPP9 was diminished during mitosis (Fig. 1b and Supplementary Fig. 1f), the protein level of MPP9 was not significantly affected, and a shifted band appeared during the G2/M phase (Fig. 1c).

Next, to observe the localization of MPP9 in detail, we used 3D-structured illumination microscopy (3D-SIM). Surprisingly, MPP9 appeared as a ring-like structure surrounding the centriole distal-end marker Centrin-3 and was colocalized with other distal-end markers, such as CP110, CEP97, and KIF24 (Fig. 1d, e and Supplementary Fig. 1g, h), and the MPP9 foci represented punctate structures were localized at the proximal parts of centrioles (Fig. 1d, e). We further imaged MPP9 along with acetylated tubulin or CEP164, markers of the centriolar barrier and distal appendages, respectively, and found that the ring-like structures formed by MPP9 were localized at the distal rims of both centrioles. Furthermore, we observed that the diameter of MPP9 was very close to that of the centriolar tubule, but smaller than that of CEP164 (Fig. 1f–i). In addition, the ring-like structures of MPP9 at the distal ends of the centrioles were also confirmed by immuno-electron microscopy (Fig. 1j, k and Supplementary Fig. 1i, j).

### MPP9 is a negative regulator of cilia formation

**Fig. 2:**
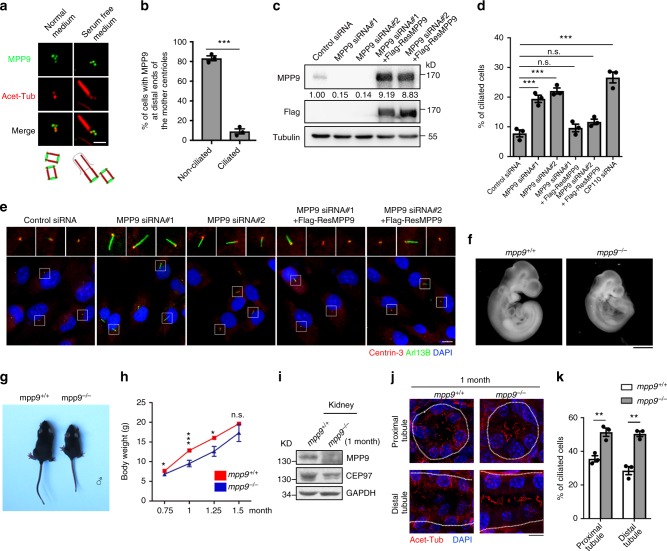
Depletion of MPP9 leads to aberrant ciliogenesis. **a** Immunostaining of acetylated-tubulin (Acet-Tub, red) and MPP9 (green) in normal and serum-free medium-treated hTERT RPE-1 cells. **b** Quantification of the percentage of cells with MPP9 at distal ends of the mother centrioles after serum starvation. **c** Immunoblots showing depletion of MPP9 by siRNAs (#1 and #2) transfection and rescue of MPP9 expression by overexpressing Flag-tagged siRNA-resistant MPP9 (Flag-ResMPP9). Tubulin was used as a loading control. Relative amounts of MPP9 were quantified and normalized to tubulin. **d** Quantification of ciliogenesis in control-siRNA, MPP9-siRNA, or CP110-siRNA, or MPP9-siRNA together with Flag-ResMPP9-rescued hTERT RPE-1 cells. **e** Immunostaining of Centrin-3 (red) and Arl13B (green) in MPP9-siRNA-treated and Flag-ResMPP9-rescued hTERT RPE-1 cells. DNA was stained with DAPI (blue). **f** Whole *mpp9*^+/+^ and *mpp9*^–/–^ embryos at embryonic day (E) 10.5. **g** The male *mpp9*^+/+^ (left) and *mpp9*^–/–^ (right) mice at 1 month after birth. **h** Line graph showing the body weight of *mpp9*^+/+^ and *mpp9*^–/–^ mice at the indicated times (*n* = 6 mice for each group). **i** Immunoblots of MPP9 and CEP97 in the kidneys of *mpp9*^+/+^ or *mpp9*^–/–^ mice at 1 month after birth. GAPDH was used as a loading control. **j** Kidneys from *mpp9* ^+/+^ and *mpp9* ^–/–^ mice (*n* = 3 mice for each group) at 1 month were stained with Acet-Tub (red) and DAPI (DNA; blue). White dashed lines represent the border of each renal tubule. **k** The percentage of ciliated cells from **j**. For **b**, **d**, **h**, and **k**, bars represent the means ± S.E.M for three independent experiments. n.s., not significant, *p < 0.05, **p < 0.01, ***p < 0.001, as determined by unpaired two-tailed Student’s *t*-test (**b**, **h**, and **k**), one-way ANOVA analysis (**d**). Scale bars: 1 mm (**f**); 5 μm (**e** main, **j**); 1 μm (**a**)

Consistent with the decrease in MPP9 protein level upon serum starvation (Fig. 1c), MPP9 was hardly detected at the distal end of the mother centriole following serum starvation in hTERT RPE-1 cells (Fig. 2a, b). To determine whether MPP9 affects primary cilium formation, we first knocked down MPP9 expression in hTERT RPE-1 cells (Supplementary Fig. 2a, b), and labeled the cells using the ciliary membrane marker, Arl13B. Surprisingly, depletion of MPP9 by two different siRNAs significantly promoted cilia formation even in the absence of serum starvation (Fig. 2c–e), and this phenotype was rescued by overexpressing siRNA-resistant MPP9 (Fig. 2c–e). Similar results were obtained in MPP9-depleted NIH-3T3 cells (Supplementary Fig. 2c), another ciliated cell line^21^, supporting the suppressor function of MPP9 in cilia formation. We further excluded the possibility that ciliogenesis was caused by cell-cycle perturbation or cell-cycle exit, as depletion of MPP9 did not affect cell-cycle process in hTERT RPE-1 cells based on flow cytometry analysis, Ki67 labeling, and the ciliary length measurement (Supplementary Fig. 2d-f). In addition, neither extra-long centrioles nor abnormal centriole numbers were detected in the non-ciliated U2OS cells after depleting MPP9 (Supplementary Fig. 2g-i).

To further elucidate the function of MPP9 in vivo, we generated *mpp9* knockout mice using the CRISPR/Cas9 apparoches^22^. After sequencing analysis, two different single base pair (1 bp) insertion mutants within the gRNA target site were detected (Supplementary Fig. 2j). Immunoblotting indicated that the band representing MPP9 was absent in brain tissue from knockout mice (Supplementary Fig. 2j). *mpp9* knockout mice showed abnormal development and a twisted body axis at midgestation (Fig. 2f). A decreased body weight phenotype was also found in the *mpp9* knockout mice compared with wild-type mice around 1 month after birth (Fig. 2g, h).

Next, we focused on the cilia-rich tissues, such as the kidney, in the *mpp9* knockout mice. Strikingly, an increased percentage of ciliated cells in the proximal and distal tubules was observed in the kidneys of *mpp9* knockout mice at 1 and 4 months after birth (Fig. 2i–k and Supplementary Fig. 2k-m), consistent with our observations in MPP9-depleted hTERT RPE-1 cells (Fig. 2c–e). The mouse embryonic fibroblasts (MEFs) isolated from *mpp9* knockout mice also showed a higher percentage of ciliation (Supplementary Fig. 2n-p), which also phenocopied that of the MPP9 siRNA-treated hTERT RPE-1 cells (Fig. 2c–e).

Together, these results suggest that MPP9 functions as a negative regulator of cilia formation both in cell lines able to form cilia and the mouse kidney.

### MPP9 interacts with KIF24 and the CP110-CEP97 complex

**Fig. 3:**
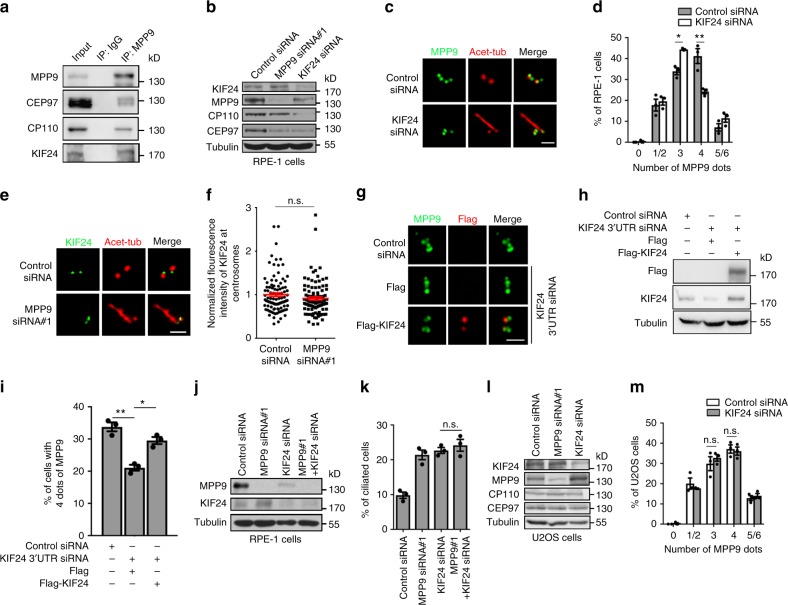
KIF24 recruits MPP9 at the distal end of the mother centriole in hTERT RPE-1 cells. **a** Lysates from hTERT RPE-1 cells were subjected to immunoprecipitation (IP) with anti-MPP9 antibody, and immunoblotting with the indicated antibodies. **b** Immunoblots showing depletion of MPP9 or KIF24 by siRNA in hTERT RPE-1 cells. Tubulin was used as a loading control. **c** Immunostaining of acetylated-tubulin (Acet-Tub, red) and MPP9 (green) in the control- or KIF24-siRNA-treated hTERT RPE-1 cells. **d** Quantification of the percentage of cells with the indicated number of MPP9 dots from **c**. **e** Immunostaining of Acet-Tub (red) and KIF24 (green) in the control- or MPP9-siRNA-treated hTERT RPE-1 cells. **f** Quantification of the fluorescence intensity of KIF24 at centrosomes from **e** (*n* = 100 cells for each group). **g** Immunostaining of MPP9 (green) and Flag (red) in KIF24-3’UTR siRNA-treated or/and Flag-KIF24-rescued hTERT RPE-1 cells. **h** Immunoblots showing depletion of KIF24 by 3’UTR siRNA transfection or/and rescue of KIF24 expression by overexpression of Flag-KIF24. Tubulin was used as a loading control. **i** Quantification of the percentage of cells with 4-dot MPP9 in control-, KIF24-3’UTR siRNA, or/and Flag-KIF24-rescued hTERT RPE-1 cells. **j** Immunoblots showing depletion of MPP9 or/and KIF24 by siRNA in hTERT RPE-1 cells. Tubulin was used as a loading control. **k** Quantification of ciliogenesis in control-, MPP9-, or/and KIF24-siRNA-treated hTERT RPE-1 cells. **l** Immunoblots showing depletion of MPP9 or KIF24 by siRNA in U2OS cells. Tubulin was used as a loading control. **m** Quantification of the percentage of cells with the indicated number of MPP9 dots in control- and KIF24-siRNA-treated U2OS cells. For **d**, **f**, **i**, **k**, and **m**, bars represent the means ± S.E.M for three independent experiments. n.s., not significant, *p < 0.05, **p < 0.01, as determined by unpaired two-tailed Student’s *t*-test (**d**, **f**, **k**, **m**) and one-way ANOVA analysis (**i**). Scale bars: 1 μm (**c**, **e**, **g**)

To reveal the mechanism underlying MPP9-mediated negative regulation of cilia formation, we screened for MPP9-interacting proteins by mass spectrometry analysis in HEK293T cells overexpressing Flag-MPP9, from which we identified several negative regulators of ciliogenesis, such as CEP97, CP110, and KIF24 (Supplementary Fig. 3a). Immunoprecipitation confirmed that MPP9 interacted with CEP97, CP110, and KIF24 (Fig. 3a and Supplementary Fig. 3b). After purification and incubation with these proteins in vitro, we found that glutathione S-transferase (GST)-tagged MPP9 could be pulled down by maltose binding protein (MBP)-fused CEP97 and KIF24, but not CP110 (Supplementary Fig. 3c-e). Next, we further mapped the domains of MPP9 that mediated these interactions by pull-down assays using purified proteins in vitro and by yeast two-hybrid assays. The 401-800 aa portion of MPP9 bound to CEP97 (Supplementary Fig. 3f), and the 451–500 aa portion of MPP9 was required and sufficient to bind to CEP97 (Supplementary Fig. 3g-i); while MPP9 mainly interacted with KIF24 through the 801–1031 aa portion of MPP9 (Supplementary Fig. 3j). We then performed the reciprocal experiment to determine the regions of CEP97 and KIF24 participating in MPP9 binding. The 587–865 aa portion of CEP97 mainly bound to MPP9 (Supplementary Fig. 3k, l) and the 478–709 aa portion of KIF24 was essential for association with MPP9 (Supplementary Fig. 3m, n). Collectively, these results indicate that MPP9 is most likely to interact with KIF24 and CEP97 through distinct domains in a direct manner.

### KIF24 recruits MPP9 to the distal end of mother centriole

To investigate the relationship between MPP9 and KIF24, we knocked down KIF24 or MPP9 in hTERT RPE-1 cells by siRNA. After KIF24 depletion, both the total protein level and the distal end localization of MPP9 at the mother centriole were decreased (Fig. 3b–d). In contrast, neither the protein level nor the centrosome localization of KIF24 changed following MPP9 depletion (Fig. 3b, e, f). Overexpression of KIF24 could rescue the localization of MPP9 at the distal ends of the mother centrioles in KIF24-depleted cells (Fig. 3g–i). Furthermore, compared with depletion of KIF24 alone, no significant increase in cilia formation was detected after simultaneously depleting KIF24 and MPP9 (Fig. 3j, k), suggesting that MPP9 and KIF24 share a common pathway in the regulation of cilia formation. Interestingly, we did not detect any change in the centrosome localization of MPP9 in the KIF24-depleted U2OS cells (Fig. 3l, m). Together, these data indicate that KIF24 is only required for recruiting MPP9 to the distal end of the mother centriole in cells that can form cilia.

### MPP9 inhibits ciliogenesis via recruiting CP110 and CEP97

**Fig. 4:**
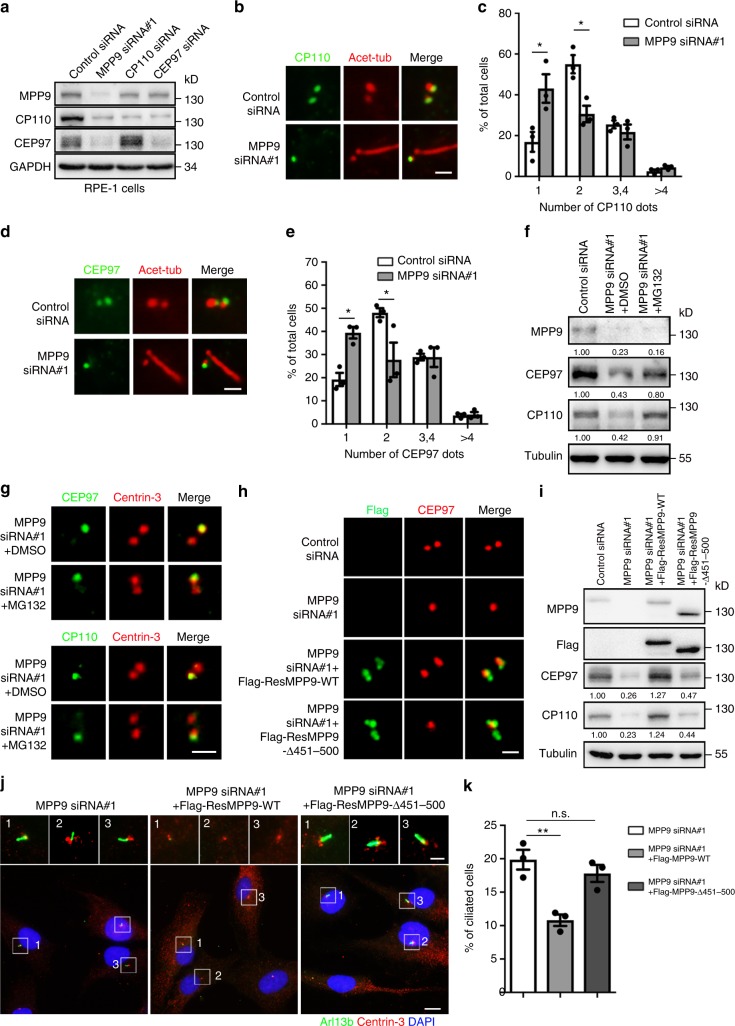
MPP9 recruits CEP97 and CP110 at the distal end of the mother centriole in hTERT RPE-1 cells. **a** Immunoblots showing depletion of MPP9, CP110, or CEP97 by siRNA in hTERT RPE-1 cells. GAPDH was used as a loading control. **b** Immunostaining of acetylated-tubulin (Acet-Tub, red) and CP110 (green) in the control- or MPP9-siRNA-treated hTERT RPE-1 cells. **c** Quantification of the percentage of cells with the indicated number of CP110 dots from **b**. **d** Immunostaining of Acet-Tub (red) and CEP97 (green) in the control- or MPP9-siRNA-treated hTERT RPE-1 cells. **e** Quantification of the percentage of cells with the indicated number of CEP97 dots from **d**. **f** Immunoblots showing expression of the indicated proteins in MPP9-depleted hTERT RPE-1 cells after treatment with DMSO or MG132 for 4 h. Tubulin was used as a loading control. Relative amounts of MPP9, CEP97, and CP110 were quantified and normalized to tubulin. **g** Immunostaining of Centrin-3 (red) and CEP97 (green, upper) or CP110 (green, lower) after treating with DMSO or MG132 for 4 h in MPP9-depleted hTERT RPE-1 cells. **h** Immunostaining of CEP97 (red) and Flag-siRNA-resistant MPP9 (Flag-ResMPP9, green) or Flag-ResMPP9-△451-500 (green) in MPP9-depleted hTERT RPE-1 cells. **i** Immunoblots showing expression of the indicated proteins in hTERT RPE-1 cells transfected with MPP9-siRNA and Flag-siRNA-resistant MPP9 wild-type (Flag-ResMPP9-WT) or lacking 451–500 aa mutant (Flag-ResMPP9-△451–500). Tubulin was used as a loading control. Relative amounts of CEP97 and CP110 were quantified and normalized to tubulin. **j** Immunostaining of Centrin-3 (red) and Arl13B (green) after transfection of Flag-ResMPP9-WT or Flag-ResMPP9-△451–500 in MPP9-depleted hTERT RPE-1 cells. DNA was stained with DAPI (blue). **k** Quantification of ciliogenesis in MPP9-depleted hTERT RPE-1 cells overexpressing Flag-ResMPP9-WT or Flag-ResMPP9-△451–500. For **c**, **e**, **k**, bars represent the means ± S.E.M for three independent experiments. n.s., not significant, *p < 0.05, **p < 0.01, as determined by unpaired two-tailed Student’s *t*-test (**c**, **e**), and one-way ANOVA analysis (**k**). Scale bars: 5 μm (**j** main); 1 μm (**b**, **d**, **g**, **h**, **j** insets)

Next, we examined the relationship among MPP9, CEP97, and CP110. Both the protein levels and the number of centrosomal dots representing CEP97 and CP110 localization at the centrosomes were significantly decreased in MPP9-depleted hTERT RPE-1 cells (Fig. 4a–e). Similarly, in *mpp9* knockout MEF cells, CEP97 and CP110 also demonstrated the deficiency in centrosome localization (Supplementary Fig. 4a-d), whereas depletion of CEP97 or CP110 hardly affected either the total protein level of MPP9 or its distal-end localization at the mother centrioles (Fig. 4a and Supplementary Fig. 4e). Addition of MG132, a proteasome inhibitor, rescued the protein level, but not the mother centriole localization of CP110-CEP97 complex in MPP9-depleted hTERT RPE-1 cells (Fig. 4f, g and Supplementary Fig. 4f), indicating that MPP9 prefers to recruit rather than to stabilize the CP110-CEP97 complex at the mother centrioles. Compared with wild-type MPP9, a MPP9 mutant lacking the binding region (451–500 aa) for CEP97 could hardly rescue the mother centriole localization of CEP97, the protein levels of CEP97 and CP110, and the percentage of ciliation in the MPP9-depleted hTERT RPE-1 cells (Fig. 4h–k and Supplementary Fig. 4g, h), strongly indicating that the 451–500 aa portion of MPP9 is required for the recruitment of CEP97 to the distal ends of the mother centrioles in ciliated cells.

When knocking down both MPP9 and CP110 or CEP97 in hTERT RPE-1 cells, the percentage of ciliated cells was not remarkably increased compared with that of the CP110 or CEP97 knockdown alone (Supplementary Fig. 4i, j), indicating that MPP9, CP110, and CEP97 likely function in the same pathway for regulating cilia formation. However, neither the protein level nor the centrosome localization of CEP97 or CP110 was affected in MPP9-depleted U2OS cells (Supplementary Fig. 4k-m).

Taken together, these data suggest that MPP9 negatively regulates cilia formation by recruiting the CP110-CEP97 complex at the distal end of the mother centriole in cells able to form cilia.

### TTBK2 phosphorylates MPP9 at mother centriole distal-end

**Fig. 5:**
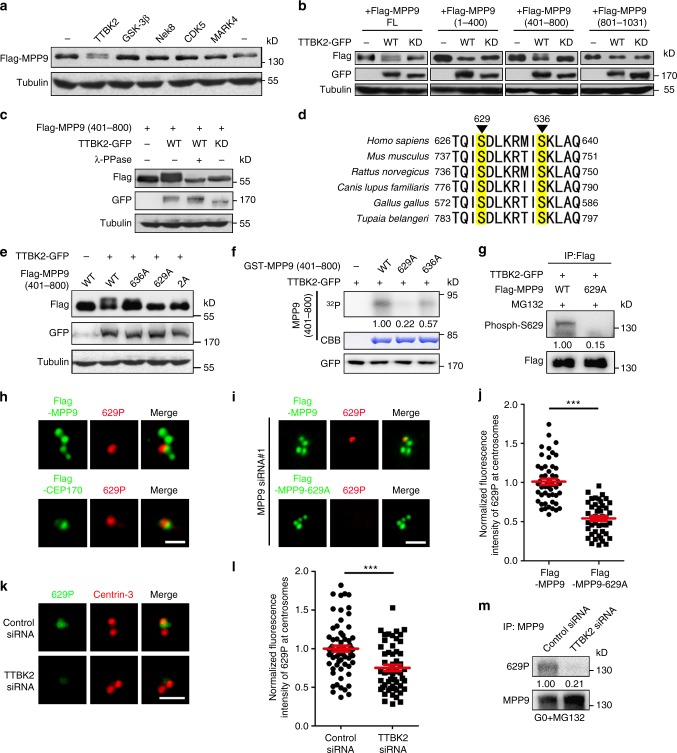
TTBK2 phosphorylates MPP9 mainly at S629 site. **a** Immunoblots of lysates from HEK293T cells co-overexpressing Flag-MPP9 and the indicated cilia-related kinase. Tubulin was used as a loading control. **b** Immunoblots of lysates from HEK293T cells co-overexpressing Flag-tagged MPP9 full-length (FL) or the indicated MPP9 truncation mutants with TTBK2-GFP-WT or TTBK2-GFP-KD. Tubulin was used as a loading control. WT, wild-type; KD, kinase-dead. **c** Immunoblots of lysates from HEK293T cells co-overexpressing Flag-tagged MPP9 (401–800 aa) and TTBK2-GFP-WT or TTBK2-GFP-KD after treatment with λ-PPase. Tubulin was used as a loading control. **d** Mass spectrometric analysis showing phosphorylation sites of MPP9 at S629 and S636. **e** Immunoblots of lysates from HEK293T cells co-overexpressing TTBK2-GFP and Flag-MPP9 (401–800 aa)-WT, or the indicated unphosphorylatable mutants. Tubulin was used as a loading control. **f** GST-MPP9 (401–800 aa)-WT, or the indicated mutants were subjected to a TTBK2 kinase assay in vitro followed by autoradiography. Coomassie blue (CBB) staining showed GST-tagged proteins. Relative amounts of phosphorylated MPP9 signals were quantified. **g** Lysates of HEK293T cells co-overexpressing Flag-tagged MPP9-WT or the S629A mutant and TTBK2-GFP were subjected to immunoprecipitation (IP) and immunoblotting with anti-Flag and anti-Phospho-S629 antibodies. Relative amounts of S629-phosphorylated MPP9 were quantified. **h** Immunostaining of S629-phosphorylated MPP9 (red) and Flag in Flag-MPP9 (green, upper) or Flag-CEP170 (green, lower) overexpressing hTERT RPE-1 cells. **i** Immunostaining of S629-phosphorylated MPP9 (red) and Flag in Flag-MPP9 (green) or Flag-MPP9–629A (green) overexpressing hTERT RPE-1 cells after depleting MPP9. **j** Quantifications of S629-phosphorylated MPP9 signals in **i** (*n* = 50 cells for each group). **k** Immunostaining of S629-phosphorylated MPP9 (green) and Centrin-3 (red) in control- or TTBK2-siRNA transfected hTERT RPE-1 cells. **l** Quantifications of S629-phosphorylated MPP9 signals in **k** (*n* = 50 cells for each group). **m** Lysates of control-siRNA or TTBK2-siRNA transfected hTERT RPE-1 cells after serum starvation were subjected to immunoprecipitation and immunoblotting with the indicated antibodies. Relative amounts of S629-phosphorylated MPP9 were quantified and normalized to MPP9. For **j**, **l**, bars represent the means ± S.E.M for three independent experiments. ***p < 0.001, as determined by unpaired two-tailed Student’s *t*-test. Scale bars: 1 μm (**h**, **i**, **k**)

Since many protein kinases, such as TTBK2, MARK4, and GSK3β, are localized to the basal body or the cilia and serve to regulate cilia assembly during the early steps^17,23,24^, we determined whether the change in localization and protein level of MPP9 after serum starvation (Figs. 1c, 2a) were induced by phosphorylation. We first co-overexpressed MPP9 with different ciliary related kinases in HEK293T cells (Fig. 5a). A shifted band representing MPP9 appeared when wild-type TTBK2, but not kinase-dead TTBK2, was co-overexpressed with MPP9 (Fig. 5a, b). The middle portion of MPP9 (401–800 aa) was further confirmed to be responsible for the band shift (Fig. 5b), and the band was observed to diminish after treatment with lambda protein phosphatase (λ-PPase, Fig. 5c), suggesting that the middle region of MPP9 is phosphorylated by TTBK2.

Using mass spectrometry analysis, we further identified two evolutionary conserved sites at serine 629 and 636 within the middle region of MPP9 that were specifically phosphorylated by TTBK2 (Fig. 5d). We then constructed unphosphorylatable mutants that encoded alanine instead of serine at positions 629 or/and 636 (S629A, S636A, S629/S636-2A). The shifted bands of MPP9-S629A and MPP9-S629/S636-2A were hardly detected in cells co-overexpressing TTBK2 and MPP9 (Fig. 5e). Also, the S629A mutant almost abolished the phosphorylation of MPP9 by TTBK2 in the kinase assay in vitro (Fig. 5f), suggesting that S629 is the main site for MPP9 phosphorylation by TTBK2. We then raised a phospho-MPP9 antibody against serine 629, and confirmed the specificity of the antibody by peptide competition assays (Supplementary Fig. 5a-d). A band was detected only in wild-type but not the S629A mutant MPP9 with the phospho-MPP9 antibody in the cells co-overexpressing TTBK2 and MPP9 (Fig. 5g). Immunostaining with the phospho-MPP9 antibody showed that only one of the four MPP9 dots localized at the centrosomes was stained, which was localized near the mother centriole appendages marker CEP170 (Fig. 5h); compared with wild-type MPP9, MPP9-S629A was hardly detected at the distal ends of the mother centrioles using the phospho-MPP9 antibody after knockdown of endogenous MPP9 (Fig. 5i, j). Furthermore, loss of TTBK2 significantly weakened the centrosome localization and protein level of S629-phospho MPP9 (Fig. 5k–m). Thus, we conclude that serine 629 of MPP9 is phosphorylated by TTBK2, and this process mainly presents at the distal ends of the mother centrioles.

### Phosphorylation of MPP9 promotes its degradation through UPS

**Fig. 6:**
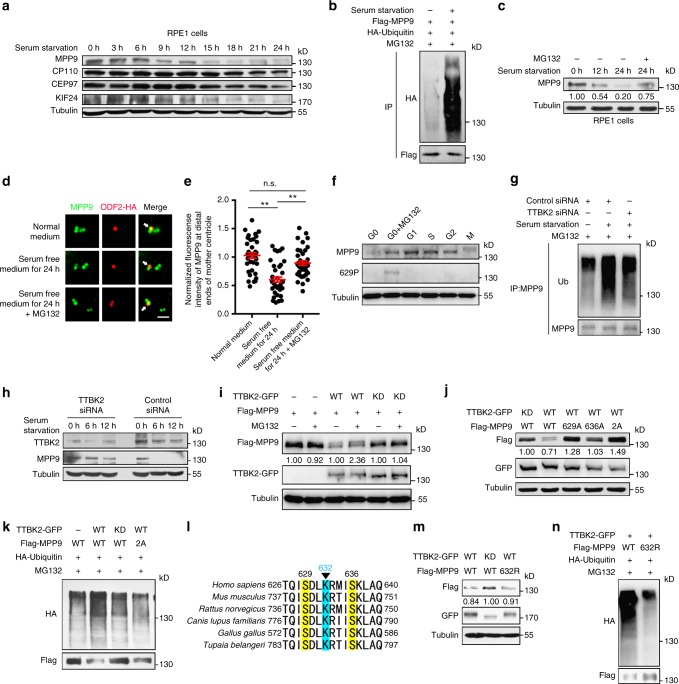
Phosphorylation of MPP9 by TTBK2 promotes UPS-mediated degradation of MPP9. **a** Immunoblots of lysates from hTERT RPE-1 cells after serum starvation. Tubulin was used as a loading control. **b** Immunoblots following immunoprecipitation (IP) with an anti-Flag antibody using lysates from hTERT RPE-1 cells overexpressing the indicated proteins. **c** Immunoblots of MPP9 after serum starvation in hTERT RPE-1 cells. Tubulin was used as a loading control. Relative amounts of MPP9 were quantified and normalized to tubulin. **d** Immunostaining of MPP9 (green) and ODF2-HA (red) in hTERT RPE-1 cells. Arrows, the distal ends of the mother centrioles. **e** Quantification of the fluorescence intensity of MPP9 at the distal ends of the mother centrioles from **d** (*n* = 40 cells for each group). **f** Immunoblots of MPP9 and S629-phosphorylated MPP9 in cycling hTERT RPE-1 cells. **g** Immunoblots following immunoprecipitation with an anti-MPP9 antibody using lysates from control- or TTBK2-siRNA-treated hTERT RPE-1 cells. **h** Immunoblots of TTBK2 and MPP9 after serum starvation in control- or TTBK2-siRNA-treated hTERT RPE-1 cells. Tubulin was used as a loading control. **i** Immunoblots of lysates from HEK293T cells co-overexpressing the indicated proteins. Tubulin was used as a loading control. Relative amounts of Flag-tagged MPP9 were quantified and normalized to tubulin. WT, wild-type; KD, kinase-dead. **j** Immunoblots of lysates from HEK293T cells co-overexpressing the indicated proteins. Tubulin was used as a loading control. Relative amounts of Flag-MPP9 were quantified and normalized to tubulin. **k** Immunoblots following immunoprecipitation with an anti-Flag antibody using lysates from HEK293T cells overexpressing the indicated proteins. **l** Mass spectrometry analysis showing the ubiquitination of MPP9 at K632 (blue). Yellow, phosphorylation sites. **m** Immunoblots of lysates from HEK293T cells co-overexpressing Flag-tagged MPP9-WT or the K632R mutant with TTBK2-GFP-WT or TTBK2-GFP-KD. Tubulin was used as a loading control. Relative amounts of Flag-MPP9 were quantified and normalized to tubulin. **n** Immunoblots following immunoprecipitation with an anti-Flag antibody using lysates from HEK293T cells overexpressing the indicated proteins. For **e**, bars represent the means ± S.E.M for three independent experiments. n.s., not significant, **p < 0.01, as determined by one-way ANOVA analysis. Scale bar: 1 μm (**d**)

As MPP9 negatively regulates cilia formation (Fig. 2), we determined whether MPP9 was decreased during ciliogenesis. Similar to the protein levels of CP110, CEP97, and KIF24, which decreased continuously during ciliogenesis in hTERT RPE-1 cells^25,26^ (Fig. 6a), the protein level of MPP9 was dramatically decreased after a 6-h serum withdrawal (Fig. 6a), while the transcription level of *mpp9* did not change (Supplementary Fig. 6a). Interestingly, no remarkable alteration in MPP9 protein level was observed in serum-starved HeLa cells (Supplementary Fig. 6b). As such, these observations further confirmed that MPP9 functions as a negative regulator of cilia formation in ciliated cells. Subsequently, since UPS-mediated protein degradation is required for ciliogenesis^25,27^, we asked whether the decrease in MPP9 also depends on this degradation pathway. Strikingly, the ubiquitination level of MPP9 significantly increased after serum starvation in hTERT RPE-1 cells (Fig. 6b). When using the proteasome inhibitor MG132 to treat cells after serum withdrawal, the reduction of the MPP9 protein level and localization at the distal ends of the mother centrioles were notably blocked (Fig. 6c–e), suggesting that MPP9 undergoes UPS-mediated degradation during ciliogenesis.

Next, we examined whether TTBK2 regulates the degradation of MPP9. Although the protein level of MPP9 decreased in G0 phase (Fig. 1c), the band of S629-phospho MPP9 could only be detected in that stage (Fig. 6f). Meanwhile, the protein level of TTBK2 increased after serum starvation in hTERT RPE-1 cells, but not in HeLa cells (Supplementary Fig. 6c). Loss of TTBK2 remarkably decreased the ubiquitination level of MPP9, and enhanced the stability of MPP9 in serum-starved hTERT RPE-1 cells (Fig. 6g, h). Furthermore, the protein level of wild-type MPP9, but not the unphosphorylatable mutants, was decreased when co-overexpressed with TTBK2, and this decrease was partially rescued by treatment with MG132 (Fig. 6i, j). When blocking protein synthesis using cycloheximide (CHX), the protein level of MPP9 decreased faster in cells stably expressing MPP9 after transfection with wild-type TTBK2 compared with that with kinase-dead TTBK2. Similarly, unphosphorylatable mutants of MPP9 degraded slower than wild-type MPP9 when co-overexpressing wild-type TTBK2 (Supplementary Fig. 6d-e). Additionally, in vivo ubiquitin assays showed that expression of either the kinase-dead TTBK2 or the MPP9-S629/S636-2A mutant significantly decreased the ubiquitination level of MPP9 (Fig. 6k). These results suggest that MPP9 phosphorylation by TTBK2 is required for UPS-mediated degradation of MPP9.

We further determined the ubiquitination sites of MPP9 by mass spectrometry analysis. When activating TTBK2, one ubiquitination site of MPP9, lysine 632, which is near the phosphorylation sites, was identified (Fig. 6l). Similar to the unphosphorylatable mutants, non-ubiquitylatable mutant of MPP9, with lysine 632 mutated to arginine (K632R), also showed increased protein stability and lower ubiquitination level after TTBK2 overexpression (Fig. 6m, n, and Supplementary Fig. 6f), which further confirms that TTBK2-mediated phosphorylation of MPP9 promotes MPP9 degradation via the UPS pathway.

### Phospho-MPP9 promotes ciliogenesis by removing CP110-CEP97

**Fig. 7:**
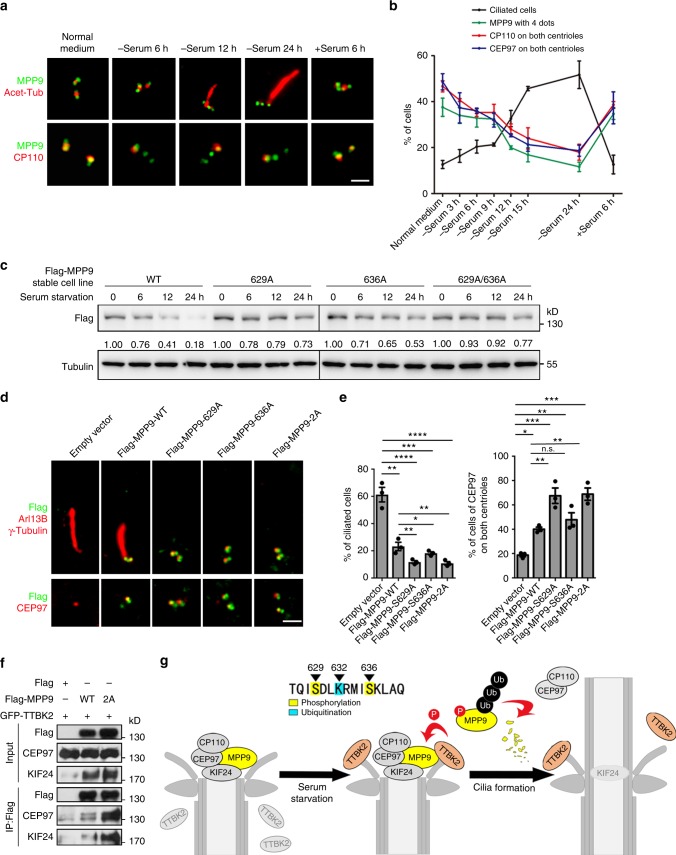
MPP9 facilitates the removal of the CEP97-CP110 complex from centrosomes after phosphorylation. **a** Immunostaining of MPP9 (green) and acetylated-tubulin (Acet-tub, red) or CP110 (red) in hTERT RPE-1 cells after serum starvation and serum re-addition. **b** Line graph showing the percentage of ciliated hTERT RPE-1 cells, the cells presenting with 4 dots of MPP9, and cells with CP110 or CEP97 on both centrioles. **c** Immunoblots of lysates from hTERT RPE-1 cells stably overexpressing Flag-tagged MPP9 wild-type (WT) or the indicated unphosphorylatable mutants after serum starvation. Tubulin was used as a loading control. Relative amounts of MPP9 were quantified and normalized to tubulin. **d** Immunostaining of Arl13B (red, upper) and γ-tubulin (red, upper) or CEP97 (red, lower) in hTERT RPE-1 cell lines stably overexpressing Flag-tagged MPP9 or the indicated unphosphorylatable mutants (green). 2, 629, and 636 A. **e** Quantification of the percentage of cells with cilia (left) and cells with CEP97 dots on both centrioles (right) from **d**. **f** Immunoblots following immunoprecipitation (IP) with an anti-Flag antibody using lysates from HEK293T cells overexpressing the indicated proteins. **g** Schematic model depicting the function of MPP9 in cilia formation control. After serum starvation, MPP9 is phosphorylated by TTBK2 at S629 and S636, which promotes its ubiquitination at K632 and degradation via UPS. Subsequently, the degradation of MPP9 causes the removal of CEP97 and CP110 from the distal end of the mother centriole and initiates cilia formation. For **b**, **e**, bars represent the means ± S.E.M for three independent experiments. n.s., not significant, *p < 0.05, **p < 0.01, ***p < 0.001, ****p < 0.0001, as determined by one-way ANOVA analysis (**e**). Scale bars: 1 μm (**a**, **d**)

Finally, we investigated the relationship between the localization of MPP9 at the centrosome and cilia formation. In the serum-contained medium, only about 10% of hTERT RPE-1 cells were ciliated, and the percentage of ciliated cells increased slowly at the beginning of serum withdrawal (0–9 h) (Fig. 7a, b). During this stage, about 50% of cells demonstrated the 4-dots pattern of MPP9 localization at the centrosome (Fig. 7a, b). Strikingly, the number of ciliated cells rapidly increased from 9 to 24 h following serum withdrawal, which was notably accompanied by the disappearance of MPP9 from the distal ends of the mother centrioles/basal bodies (Fig. 7a, b). This observation was consistent with the immunoblotting results indicating a dramatically reduced MPP9 protein level during this time (Fig. 6a). Six hours after re-introducing serum to the cells that had been serum starved for 24 h, most cilia had disassembled, and MPP9 was relocated to the distal ends of the mother centrioles/basal bodies (Fig. 7a, b). Similarly, CP110 and CEP97 were also displaced from the distal ends of the mother centrioles/basal bodies upon serum withdrawal and recovered following re-introduction of serum (Fig. 7a, b). Therefore, the centrosome localization of MPP9, similar to those of CP110 and CEP97, is inversely correlated with cilia formation.

As the level of MPP9 is regulated by phosphorylation-mediated degradation (Fig. 6), we investigated whether phosphorylation of MPP9 could control cilia formation. When compared with wild-type MPP9, the degradation of unphosphorylatable mutants of MPP9 (S629A, S636A, S629/S636-2A) was blocked after serum starvation (Fig. 7c), which resulted in an increase in the distal end localization of MPP9 at the mother centriole (Fig. 7d), and also showed significant repression of cilia formation (Fig. 7d, e). Moreover, the non-ubiquitylatable mutant of MPP9 (K632R), which demonstrated suppressed phosphorylation-mediated degradation (Fig. 6m, n), also showed similar degradation deficiency and reduction in ciliated cells (Supplementary Fig. 7a-c). Thus, we conclude that phosphorylation of MPP9 promotes its removal from the distal end of the mother centriole and is required for effective ciliogenesis.

Next, we investigated whether phosphorylation of MPP9 affects ciliogenesis by promoting the removal of the CP110-CEP97 complex from the distal end of the mother centriole. MPP9 interacted with and recruited CP110 and CEP97 to the distal end of the mother centriole in hTERT RPE-1 cells (Fig. 4). Additionally, when compared with wild-type MPP9, stably expressed unphosphorylatable MPP9 increased the percentage of cells with two CEP97 dots at the distal ends of the both centrioles in serum-starved hTERT RPE-1 cells (Fig. 7d, e) and also enhanced the interaction between CEP97 and KIF24 (Fig. 7f). These observations suggest that phosphorylation of MPP9 facilitates removal of both itself and the CP110-CEP97 complex from the distal end of the mother centriole and promotes the initiation of ciliogenesis.

## Discussion

A series of positive regulators participate in the mother centriole-to-basal body transition and regulate the initial elongation of the ciliary axoneme^9,28,29^, but negative regulators and how they cooperate with each other temporally and spatially are still poorly understood. Here we show that MPP9, recruited by KIF24, recruits the CP110-CEP97 complex to the distal ends of the mother centrioles and functions as a switch for initiating the early steps of ciliogenesis. The removal of MPP9 is induced by sensing TTBK2 signaling and subsequent UPS-mediated degradation, which leads to the displacement of CEP97 and CP110 from the distal end of the mother centriole, promoting the initiation of ciliogenesis (Fig. 7g). In interphase, MPP9 interacts with and recruits the CP110-CEP97 complex to the distal ends of the mother centrioles and blocks cilia assembly in cycling cells able to form cilia. MPP9, most likely, directly interacts with CEP97 but not CP110. Loss of MPP9 perturbs both the protein level and centrosome localization of CEP97 and CP110, but not vice versa, in ciliated hTERT RPE-1 cells, suggesting that MPP9 is an upstream regulator of the CP110-CEP97 complex (Fig. 7g). Interestingly, this recruitment mode is not detected in non-ciliated cells, such as U2OS cells, indicating that this is a cell-specific mechanism for controlling the localization and stabilization of the CP110-CEP97 complex at centrosomes. MPP9 is recruited to the distal ends of the mother centrioles by KIF24 in a cell-specific manner, similar to the recruitment of the CP110-CEP97 complex by MPP9. As KIF24 has also been reported to recruit CEP97 and CP110^12^, MPP9 may act as an adaptor protein connecting KIF24 and the CP110-CEP97 complex. Notably, our data show that the middle region of MPP9 (401–800 aa) is indispensable for its centrosome localization, distinct from its C-terminus (801–1031 aa), which interacts with KIF24. Since MPP9 is localized at both the proximal and distal ends of the centrioles, while KIF24 is only localized at the latter, it is possible that the distal end localization of MPP9 at the mother centriole mainly depends on KIF24. As a microtubule depolymerizing kinesin, KIF24 specifically controls ciliary length^12^. Whether the interaction of KIF24 with MPP9 regulates its microtubule depolymerizing activity during the cell cycle, however, still needs to be addressed.

In contrast to the function of CP110 as a negative regulator of ciliogenesis in cells, *CP110* knockout mice show deficiencies in ciliogenesis and centriolar subdistal appendages localization, suggesting CP110 also positively regulates cilia formation in vivo^30^. In *Xenopus*, CP110 also shows a dual role in cilia formation^31^. Unlike the specific role of MPP9 in ciliogenesis, CP110 may play a pivotal role in multiple aspects of centrosome functions, such as centrosome separation, centrosome duplication, and cytokinesis^32,33^. It is plausible that depletion of CP110 in vivo severely impairs the function of the centrosome, resulting in the loss of cilia.

The removal of the CP110-CEP97 complex from the centrosome is thought to be an essential event at the beginning of cilia formation^11^, but the detailed mechanisms underlying this event are still unclear. KIF24 has been reported to function as a negative regulator of cilia formation based on its ability to recruit the CP110-CEP97 complex to the basal bodies during interphase^12^. Although the protein level of KIF24 is decreased after serum starvation, the basal body localization of KIF24 shows little change under this condition, so this recruitment by KIF24 may not entirely explain how the CP110-CEP97 complex is displaced from the distal ends of the mother centrioles at the beginning of ciliogenesis. TTBK2, a cilia-related kinase, is also required for CP110 removal during the initiation of cilia formation^17^, though there is no strong evidence indicating that TTBK2 might phosphorylate one or more proteins in the KIF24-CP110-CEP97 pathway to affect the localization of CP110 at basal body. Based on the results of this current study, we have demonstrated the dual roles of MPP9 as a CP110-CEP97 complex stabilizer and a TTBK2-signaling sensor, which nicely reconciles our current understanding regarding how the CP110-CEP97 complex is removed from the basal body at the beginning of ciliogenesis. We first determine that MPP9, a KIF24-CP110-CEP97 pathway protein, could be phosphorylated by TTBK2. TTBK2 is recruited to the distal appendages of the mother centrioles after serum starvation^16,34,35^, which specifically phosphorylates one of the four MPP9 dots at the distal end of the mother centriole and accelerates MPP9 degradation at that position via the UPS pathway. UPS is one of the most important protein degradation systems and is involved in regulating ciliogenesis, as shown by recent studies^25,27^. The E3 ligases, such as pVHL, MIB-1, cdc20, FBW7, and KCTD17, are known to control cilia formation by degrading different substrates, such as PCM1, AZI1, Talpid3, Nek1, NDE1, and trichoplein^24,25,36–39^. It is possible that some cilia-related E3 ligases are recruited and activated at the basal body by TTBK2 or they might recognize the regions of MPP9 phosphorylated by TTBK2, consequently mediating the degradation of MPP9 at the beginning of ciliogenesis. Since MPP9 is required for recruiting CP110-CEP97 complex at mother centrioles (Fig. 4), the degradation of MPP9 may become one of the main events which facilitates the removal of the CP110-CEP97 complex from the mother centrioles/basal bodies, and promotes cilia formation.

Since the protein levels of CP110 and CEP97 were down-regulated after MPP9 (Fig. 4a) and/or KIF24 depletion^12^, we propose that the CP110-CEP97 complex may first be displaced from the distal end of the mother centriole and then undergo degradation. CP110 is tightly regulated by the balance between the SCF^cyclinF^ ubiquitin complex and USP33 during the S and G2/M phases^40,41^, but not in the G0 phase during which the cell can form cilia. Neurl-4, a daughter centriole-localized CP110-interacting protein, translocates to the mother centriole and acts as a ubiquitin ligase cofactor to regulate CP110 during cilia formation^42^. In contrast, a recent study has shown that CP110 isn’t degraded through the UPS pathway after serum starvation^25^. Alternatively, several studies have shown that CP110 levels are precisely regulated by microRNAs to ultimately control cilia formation during motile ciliogenesis^26,31,43^. While, as a cofactor of CP110, the mechanism underlying the degradation of CEP97 at the initial step of ciliogenesis is much less understood. Similarities and differences in the degradation mechanism between CP110 and CEP97 should be addressed in future studies.

## Methods

### Animals

All animal procedures were approved by the Committee of the College of Life Sciences, Peking University, and performed according to the University’s animal experimentation rules. *mpp9*^–/–^ mice were generated by CRISPR/Cas9-mediated genome editing^22^. In brief, superovulating female C57BL/6 mice (5–6 weeks old) were mated to C57BL/6 males, and fertilized embryos were then obtained from the oviducts. The Cas9 mRNA (from 50 to 100 ng mL^−1^) and the sgRNAs (from 25 to 50 ng mL^−1^) targeting murine *mpp9* were injected into the cytoplasm of zygotes (C57BL/6), which were transplanted into a foster female mouse (ICR). The fertilized eggs were cultured in M16 medium (Sigma-Aldrich) at 37 °C in 5% CO_2_, and all the experiments with the fertilized eggs were conducted in M2 medium (Sigma-Aldrich). Eventually, Mice were genotyped by PCR using the forward primer GCGTCGACTCAGTCAGGCTTACCTGGC and the reverse primer GCGGATCCAGACAGAGGG ATACTGTTTT, and polyacrylamide gel electrophoresis (PAGE) was used to identify *mpp9*^*–/–*^ mice^44^.

### Primary mouse fibroblasts

For the primary culture of MEF cells, embryos were harvested on days 11.5–13.5. Liver tissue was used for DNA extraction and genotyping, and other viscera and heads were removed. The embryo body was then washed three times in phosphate-buffered saline (PBS) and transferred into a P100 dish with 5 mL of 1× trypsin. Each embryo was minced for 2–3 min and placed in the dish in a 37 °C incubator. After about 15–30 min, the cells and tissue bits were transferred to a 15 mL conical tube with a 10 mL pipet. Next, the cells were pipetted several times to further dissociate the tissue, followed by the addition of 5 mL medium (DMEM + 10% FBS). Next, the cells were spun in a table-top centrifuge at 130×*g* for 5 min. The embryonic cells in the supernatant were resuspended in 10 mL fresh medium, transferred to a clean sterile P100 dish, and then incubated at 37 °C in 5% CO_2_.

### Plasmid construction

Human M-Phase Phosphoprotein 9 (MPP9; NM_022782.3 [https://www.ncbi.nlm.nih.gov/nuccore/NM_022782.3]), mouse MPP9 (NM_001277867.1 [https://www.ncbi.nlm.nih.gov/nuccore/NM_001277867.1]), and all fragments and mutants were amplified by PCR and cloned into pEGFP-N3 (Clontech Laboratories), pCMV-3 × Flag-7.1 (Sigma-Aldrich), and pGEX-6P-1 (GE Healthcare). The GFP- and Flag-tagged CP110 and CEP97 were kindly provided by Dr. Brian D. Dynlacht. The cDNA of KIF24 and TTBK2 were purchased from Vigene Biosciences, and TTBK2-D163A (kinase dead) mutant was mutated by PCR and cloned into pEGFP-N3 (Clontech Laboratories).

### Antibodies

All the antibodies used were listed in the Supplementary Table 1. Anti-KIF24 antibodies were raised against glutathione-S-transferase (GST) fusion protein containing residues 601–780 aa of human KIF24^12^. Anti-phosphorylated S629-MPP9 antibodies were raised in rabbits using a keyhole limpet hemocyanin (KLH)-conjugated ISDLKRMI(pS)KLEAQVKQ peptide, which was affinity purified on phosphorylated- and/or nonphosphorylated-epitope-bound columns by ABclonal Biotechnology.

### Cell culture and transfection

HEK293T (ATCC, CRL-11268G-1), HeLa (ATCC, CCL-2), U2OS (ATCC, HTB-96), MEF (lab-generated), and NIH-3T3 (ATCC, CRL-1658) cells were cultured in DMEM (GIBCO) supplemented with 10% FBS (GIBCO or CellMax) at 37 °C with 5% CO_2_. hTERT RPE-1 (ATCC, CRL-4000) cells were maintained in DMEM/F12 (1:1) containing 10% FBS (GIBCO) and 0.01 mg mL^−1^ hygromycin B at 37 °C with 5% CO_2_. Plasmids and siRNAs were transfected into hTERT RPE-1, HEK293T, HeLa, and U2OS cells using Lipofectamine^TM^ 2000 (Invitrogen), based on the manufacturer’s protocols.

### Cell synchronization and drug treatment

U2OS cells and hTERT RPE-1 cells were synchronized in mitosis with the addition of nocodazole for 17 h. For double-thymidine block, U2OS cells and hTERT RPE-1 cells were subjected to thymidine for 18 h, followed by release for 12 h, and subsequently blocked again for 18 h. hTERT RPE-1 cells were arrested in G1 phase by treatment with mimosine for 24 h. For the G2 phase arrest, hTERT RPE-1 cells were treated with RO3306 for 20 h. For entering G0 phase, hTERT RPE-1 cells were serum starved for at least 12 h.

The drugs used were cycloheximide (CHX, 100 μg mL^−1^, Amresco), MG132 (10 mM, Sigma-Aldrich), nocodazole (100 nM, Sigma-Aldrich), thymidine (2.5 mM, Sigma-Aldrich), mimosine (400 μM, Selleck) and Ro3306 (10 μM, Selleck). To facilitate ciliation, hTERT RPE-1 cells were serum starved in DMEM/F12 (1:1) for 12–48 h.

### Generating stable cell line

Cultured hTERT RPE-1 cell lines stably expressing MPP9 or its mutants were generated as follows: MPP9 and its mutants were constructed into p3× FLAG-CMV-14 expression vector which confers resistance to G418. The transfection was performed using Fugene 6 (Promega) reagent, followed by selection with 500 μg mL^−1^ G418 for one week. Subsequently, single clones of the cells were chosen and cultured in the medium with 200 μg mL^−1^ G418 for immunoblotting and immunostaining analysis.

### Immunofluorescence and immuno-electron microscopy

For immunofluorescence, briefly, cells on coverslips were fixed in cold methanol for 10 min or 4% paraformaldehyde (PFA) with 0.1% Triton X-100 at room temperature for 20 min, then washed by PBS for three times, and blocked by PBS with 5% Bovine Serum Albumin (BSA)^45^. The primary and secondary antibodies used for immunofluorescence were shown in Supplementary Table 1. Multiplex immunofluorescence with Tyramide Signal Amplification (TSA) for cells was a methodology that enabled simultaneous detection of multiple proteins of interest in cultured cells in a stepwise fashion, regardless of the species of antibody host. Briefly, cells were fixed in pre-cooled methanol for 10 min at −20 °C. Endogenous peroxidase activity was quenched by addition of 0.3% hydrogen peroxide in PBS for 20 min at room temperature. The cells were then washed three times in PBS for 10 min each time. After blocking the cells with BSA for 30 min, cells were incubated with the primary antibody diluted in 4% BSA at 4 °C overnight followed by HRP-conjugated goat-anti-mouse or goat-anti-rabbit secondary antibody at room temperature for 30 min. Fluorophore-conjugated TSA amplification reagent (Probe Biotech Co., Ltd) diluted in 1:100 reaction buffer (Lumiffer; Probe Biotech Co., Ltd) was added to the sample and incubated for 30–120 s until the wanted signal intensity and a higher signal-to-noise ratio was achieved. An antibody stripping buffer (AbCracker; Probe Biotech Co., Ltd) was employed to eliminate the antibodies while retaining the fluorophore-TSA signal by incubation for 10–30 min at 37 °C. Then next protein was serially detected following the above methodology.

For frozen sections, mouse tissues were dissected out from 1-month-old or 4-month-old mice, fixed in 3–4% PFA in PBS at room temperature for 2 h, further fixed at 4 °C overnight, dehydrated through a graded sucrose series, and embedded in Optimal Cutting Temperature (OCT) compound (Sakura Finetek). Cryosections were cut with cryostat (CM1850, Leica) at 10 μm, and were post-fixed in 4% PFA and permeabilized in 0.5% Triton X-100.

All the samples were observed at room temperature under a fluorescence microscope (TH4-200; Olympus) equipped with a 60 × 1.42 NA Apo oil objective lens (Olympus) or a confocal microscope (TCS SP8; Leica) equipped with a 100 × 1.4 NA objective lens (Leica). Images were acquired using DP controller software (Olympus) or Las X software (Leica). Super-resolution microscopy was performed with a three-dimensional structured illumination microscope (3D-SIM) using the N-SIM System (Nikon) equipped with a 100 × 1.49 NA Apo oil objective lens (Nikon). All images were reconstructed to maximum projections, except those referred to as 3D reconstructions, using NIS-Elements AR software (Nikon). Image processing was performed in Photoshop (CC; Adobe).

For immuno-electron microscopy, U2OS cells fixed with 4% PFA were incubated with phosphate buffer (PB; 0.2 M Na_2_HPO_4_, 0.2 M NaH_2_PO_4_; pH 7.4) plus 0.5% Triton X-100 for 5 min^46^. The primary antibody was incubated with an anti-MPP9 rabbit antibody (1:20; Sigma-Aldrich, HPA037485), and the secondary antibody was incubated with anti-rabbit IgG-gold antibody (10 nm colloidal gold; 1:10; Sigma-Aldrich, G7402). Immunogold-stained samples were imaged with a transmission electron microscope (Tecnai G2 20 Twin; FEI).

### Immunoprecipitation and pull-down assays

For immunoprecipitation, cells were collected after washing three times in cold PBS (137 mM NaCl, 2.7 mM KCl, 4.3 mM Na_2_HPO_4_, 1.4 mM KH_2_PO_4_; pH 7.4) and lysed with ELB buffer (50 mM HEPES, 250 mM NaCl, 5 mM EDTA, 0.1% NP-40, 1 mM DTT, 10% glycerol; pH 7.5, and protease inhibitors) on ice for 30 min. The lysates were centrifuged at 20,000×*g* for 20 min at 4 °C, and the supernatants were mixed with the related antibodies at 4 °C for 2 h, then incubated with Protein G Sepharose beads (GE Healthcare) for at least 2 h. After washing in ELB buffer several times, the beads were boiled at 100 °C for 10 min with SDS loading buffer. For dephosphorylation assays, cell lysates were subjected to λ PPase (NEB) for 30 min, as based on the manufacturer’s protocols.

For pull-down assays, purified GST-fused or MBP-fused proteins expressed in *E. coli* were mixed with Glutathione-Sepharose 4B beads (GE Healthcare) or Amylose Resin beads (NEB) for 2 h, respectively. After extensive washing with PBS, the beads were incubated with the supernatants from cell lysates or purified GST-tagged fusion proteins at 4 °C for at least 2 h and washed with lysis buffer. The beads were then boiled at 100 °C for 5 min in SDS loading buffer.

### Immunoblotting

For immunoblotting, samples were resolved by SDS-PAGE gels and transferred to a PVDF membrane (Millipore), which was then incubated with primary antibodies (Supplementary Table 1) and HRP-conjugated secondary antibodies (1:5000, Jackson ImmunoResearch). Representative uncropped blots are shown in Supplementary Fig 8.

### Peptide competition assays

For peptide competition assays, anti-phospho-MPP9 antibodies were pre-incubated with the indicated peptides (peptide/antibody = 5:1) for 1 h at room temperature, then performed immunoblotting or immunostaining experiments.

### Sample preparations for mass spectrometry analysis

For preparation of samples for mass spectrometric analysis, cells were lysed in ice-cold ELB buffer supplemented with protease inhibitors and phosphatase inhibitor PhosSTOP (Roche). The lysates were centrifuged, and the supernatants were subjected into immunoprecipitation at 4 °C with anti-Flag antibody coated Protein G beads for 2 h. The beads were washed three times by lysis buffer and then boiled in SDS loading buffer at 100 °C for 5 min. The samples were visualized into the SDS-PAGE gels and stained by a silver staining kit (Pierce). The distinct protein bands were subjected to tryptic digestion and analysed by Orbitrap Elite mass spectrometer (ThermoFisher Scientific) for screening interacting proteins or protein modifications.

### Yeast two-hybrid assays

MPP9 fragments were cloned into the prey vector pGBKT7 (Clontech Laboratories), full-length and/or fragments of CEP97, CP110, and KIF24 were cloned into the bait vector pGADT7. For yeast two-hybrid experiments, the prey and bait vectors were co-transformed into the AH109 yeast strain, then plated onto both double (Trp−, Leu−) and quadruple (Trp−, Leu−, His−, Ade−) selective medium for 2–4 days at 30 °C^47^.

### RNAi

The siRNA oligonucleotides used in this research were obtained from Invitrogen, and the sequences are listed in the Supplementary Table 2. The siRNA-transfected cells were analyzed 48–72 h after transfection. To generate siRNA-resistant MPP9 (resMPP9), silent mutations were introduced into the MPP9 sequence by siRNA (5′- CGCTAAAGAAATTCCATGTT-3′ was mutated to 5′-CACTCAAAAAGTTTCACGTA-3′ and 5′-GCCACCGATAACCATGTTAA-3′ was mutated to GCAACAGACAATCACGTCAA).

### Production of Cas9 mRNA and gRNA

For Cas9 mRNA and gRNA production^48^, the T7 promoter was respectively added to the Cas9 coding region and to the gRNAs template by PCR amplification, and Cas9 mRNA and gRNA were transcribed in vitro using a mMESSAGE mMACHINE T7 ULTRA kit (Life Technologies), respectively.

### Flow cytometry analysis

The cells were trypsinized, suspended in 100 mL PBS, and fixed by drop-wise addition of 1 mL cold 70% ethanol. The fixed cells were washed with PBS containing 0.1% Triton X-100 and incubated with 100 mg mL^−1^ RNase A and 10 mg mL^−1^ propidium iodide (PI) for 1 h. The cells were then analyzed using flow cytometer (FACS calibur, BD Biosciences) and CellQuest software (BD Biosciences).

### RNA isolation and qRT-PCR

TRIzol (Invitrogen) was used to extract the total RNA. Quantitative real-time PCR was performed using SYBR Green PCR Master Mix (Invitrogen) in an ABI 7300 Detection System (Applied Biosystems) as previously described^49^.

### In vitro kinase assay

TTBK2-GFP overexpressed HEK293T cells were lysed in lysis buffer (50 mM HEPES; pH 7.4, 150 mM NaCl, 1 mM EDTA, 0.5% Triton X-100, protease inhibitors). TTBK2-GFP were immunoprecipitated employing anti-GFP antibody coupled to protein G-Sepharose beads. After washing three times in lysis buffer, and once in kinase buffer without ATP (50 mM Tris; pH 7.7, 10 mM MgCl_2_, 1 mM DTT, protease inhibitors), the TTBK2-GFP beads were incubated with 2 mg recombinant purified GST-MPP9 (401–800 aa)-WT, GST-MPP9 (401–800 aa)-S629A or GST-MPP9 (401–800 aa)-S636A adding 0.5 μL 10 mCi mL^−1^ γ-[32 P] ATP with a final 100 mM ATP. Eventually, the reactions were incubated for half an hour at 30 °C.

### Measurement of the relative pixel intensity of centriolar rings

The images of the centrosomal proteins (Fig. 1f, h) were acquired by structured illumination microscopy (SIM). The relative pixel intensity of the rings formed by these proteins (Fig. 1g, i) was measured and calculated using NIS-Elements AR software (Nikon). To measure the relative pixel intensity of the ring, the distance passing through the center of a circle between two intensity maxima was employed using top-view or side-view images observed from three different directions^15^.

### Quantification of fluorescent intensity and immunoblotting bands

Fiji software (NIH) was used to measure the fluorescence intensity of centrosomal proteins. A circle was drawn surrounding the centriole to obtain the fluorescent intensity of the centrosomal proteins of interest, and the total density of centrosomal proteins and the circled region were measured. In order to reduce the background signal from adjacent regions, three adjacent circles of the same size were drawn to acquire the average intensity. The signal ratio of the circled region over the centrosomal proteins was then normalized to the average signal ratio based on the control group.

For measuring the intensity of immunoblotting bands, a rectangle was drawn surrounding the target band and the subsequent steps were similar to the aforementioned calculation of the fluorescent intensity.

### Statistical analysis

Statistical analysis was performed using Prism 5 (GraphPad Software). Student’s *t*-test was used for two groups comparing. For multiple comparisons, one-way ANOVA analysis was used. All experiments were performed three times. Asterisks denote * *p* < 0.05, ** *p* < 0.01, *** *p* < 0.001, **** *p* < 0.0001, n.s. not significant.

## Electronic supplementary material

Supplementary Information Huang et al

## Data Availability

The mass spectrometry proteomics data of MPP9 interacting proteins are available via ProteomeXchange with identifier PXD011126. Source data for figures are available from the corresponding author upon request.
