# Supplementary material for: M-Phase Phosphoprotein 9 regulates ciliogenesis by modulating CP110-CEP97 complex localization at the mother centriole
Source: Nat Commun. 2018 Oct 30;9:4511. doi: 10.1038/s41467-018-06990-9 (PMC6207757; doi:10.1038/s41467-018-06990-9)
Supplement: Supplementary file 1 — Supplementary Information Huang et al [file 41467_2018_6990_MOESM1_ESM.pdf]

Supplemental Information

M-Phase Phosphoprotein 9 regulates ciliogenesis by modulating CP110-CEP97 complex localization at the mother centriole

Huang et al.

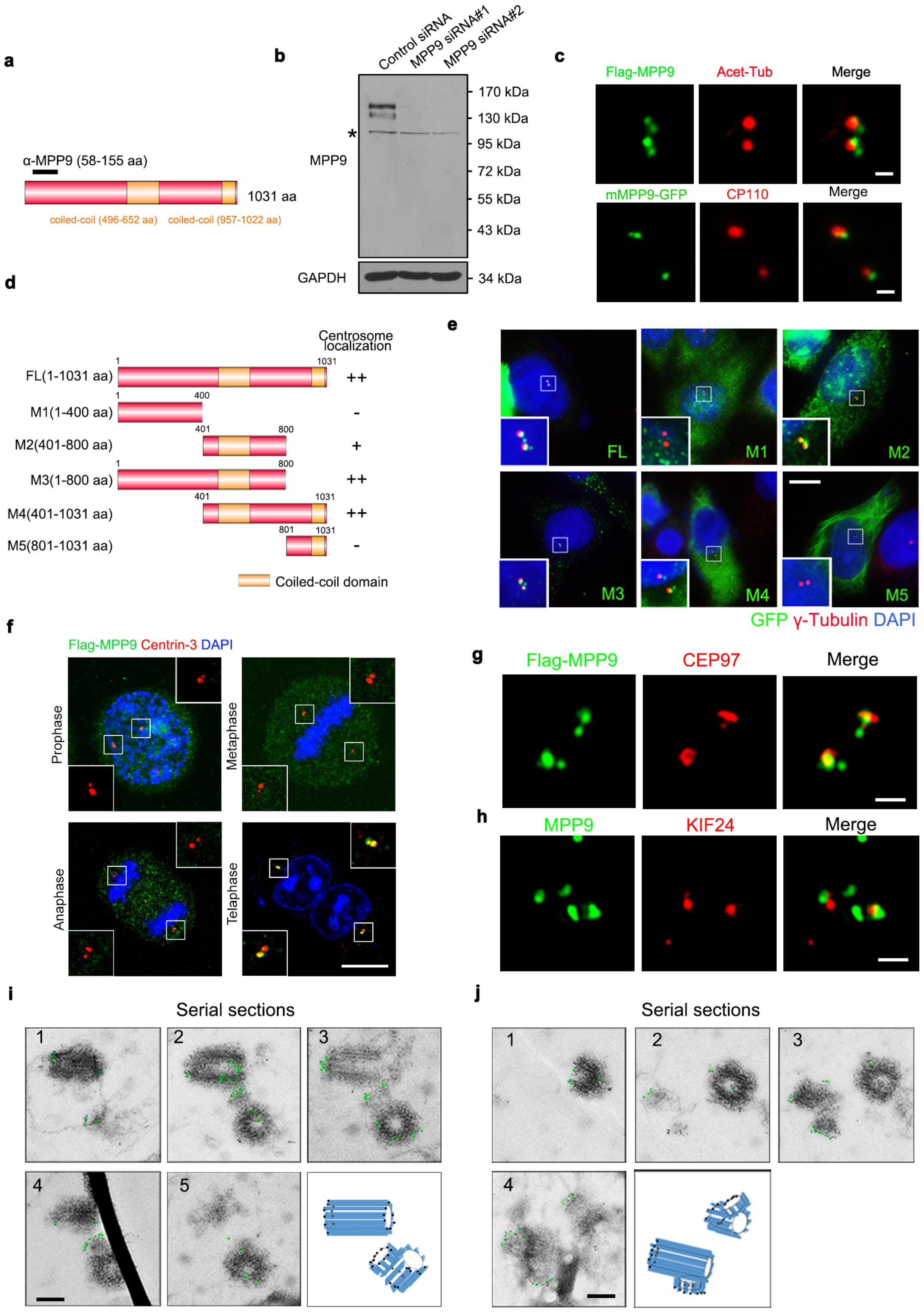

Supplementary Figure 1. Detailed localization of MPP9 at centrosomes. a Schematic of human MPP9. Coiled-coil domains, yellow; antigen, black line. **b** Immunoblots with anti-MPP9 antibody in the control- and MPP9-siRNA-treated hTERT RPE-1 cells. GAPDH was used as a loading control. The asterisk indicates a nonspecific band detected by the anti-MPP9 antibody. **c** Immunostaining of acetylated-tubulin (Acet-tub, red, upper) and Flag (green, upper) in the Flag-MPP9 overexpressing hTERT RPE-1 cells, and CP110 (red,

lower) in the GFP-tagged mouse MPP9 (mMPP9, green, lower) overexpressing hTERT RPE-1 cells. **d** Schematic of human MPP9 and its truncated mutants. Coiled-coil domains, yellow; centrosome localization: +, positive; –, negative; FL: full-length. **e** Immunostaining of γ-tubulin (red) in U2OS cells overexpressing GFP-tagged full-length (FL) MPP9 or its truncated mutants (green) from **d**. DNA was stained with DAPI (blue). **f** Immunostaining of Centrin-3 (red) and Flag (green) in the Flag-MPP9 overexpressing hTERT RPE-1 cells. DNA was stained with DAPI (blue). **g** Immunostaining of Flag (green) and CEP97 (red) in Flag-MPP9 transfected hTERT RPE-1 cells. **h** Immunostaining of MPP9 (green) and KIF24 (red) in the hTERT RPE-1 cells. **i, j** Serial immuno-electron microscopy images. U2OS cells were labeled with anti-MPP9 antibody followed by anti-rabbit IgG-gold (10 nm) secondary antibodies (green). Schematics of immuno-electron microscopy images are shown. Scale bars: 1 μm (**c**, **g**, **h**); 5 μm (**e, f**); 200 nm (**i**, **j**).

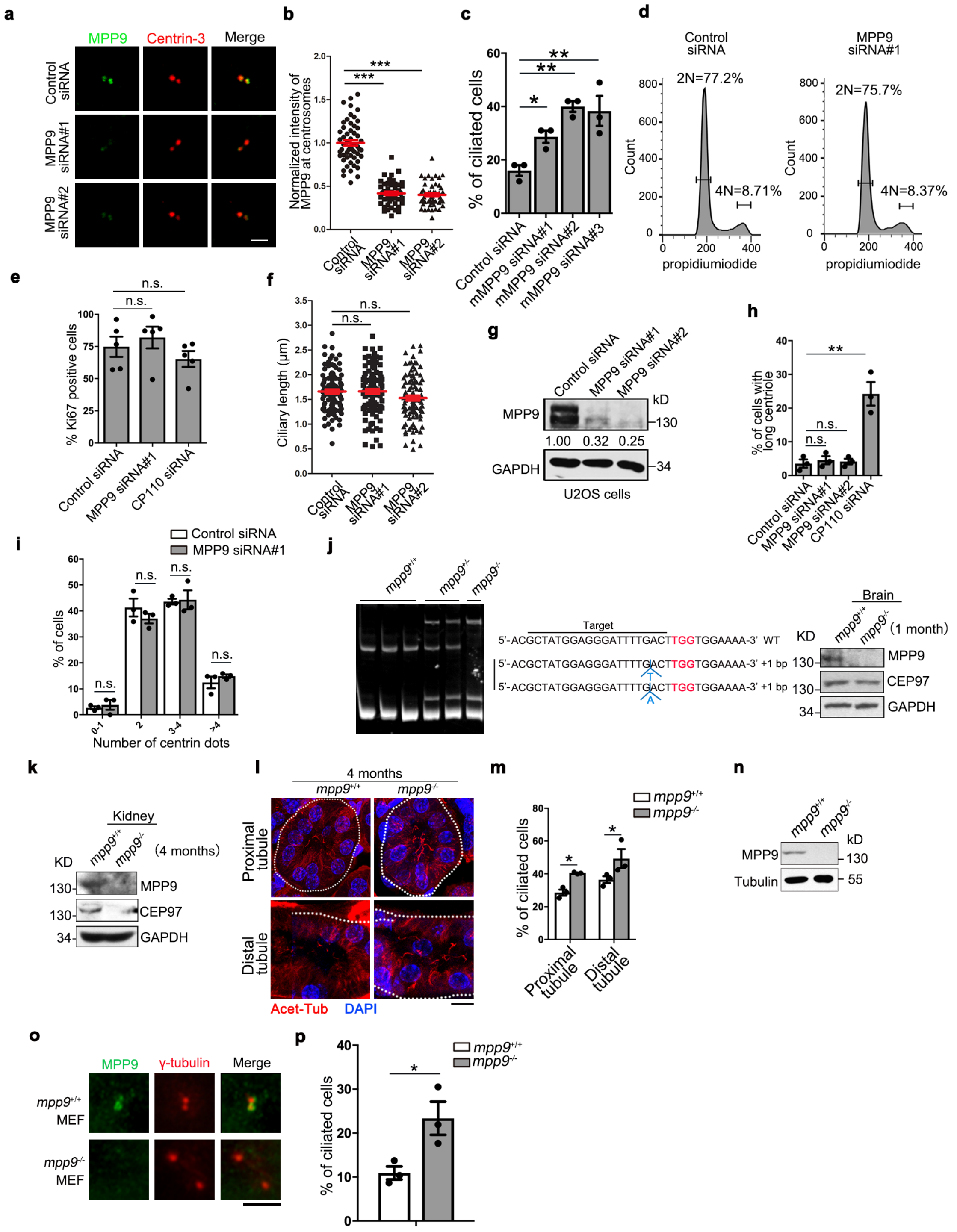

Supplementary Figure 2. Loss of MPP9 affects ciliogenesis but not cell cycle procession. a Immunostaining of MPP9 (green) and C-Nap1 (red) in the control- or MPP9-siRNA-treated hTERT RPE-1 cells. **b** Quantifications of MPP9 intensity at centrosomes from **a** (n= 50 for each group). **c** Quantification of the ciliogenesis in control- and mMPP9-siRNA-treated NIH-3T3 cells. **d** Flow cytometry analysis of control- and MPP9-siRNA-treated hTERT RPE-1 cells. **e** Quantification of Ki67-positive cells in the control-, MPP9-, or CP110-siRNA-treated hTERT RPE-1 cells. **f** Quantification of ciliary length in the control-, or MPP9-siRNA-treated hTERT RPE-1 cells (n= 100 for each group). **g** Immunoblots of MPP9 in

the control- or MPP9-siRNAs-treated U2OS cells. GAPDH was used as a loading control. Relative amounts of MPP9 were quantified and normalized to GAPDH. **h** Quantification of cells with long centrioles in the control-, MPP9-, and CP110-siRNA-treated U2OS cells. **i** Quantification of the percentage of cells with the indicated number of centrin dots in the control- and MPP9-siRNA-treated U2OS cells. **j** Based on the PAGE genotyping protocol, 15% PAGE analysis detected *mpp9*$^{+/+}$, *mpp9*$^{+/–}$ and *mpp9*$^{–/–}$ mice, as indicated. Schematic of the Cas9/sgRNA-targeting site in mouse *mpp9* is shown. The sgRNA-targeting sequence is underlined, and the PAM sequence is labeled in red. Sequencing results revealed mice harboring two kinds of single base (1 bp) insertions (shown in blue). Total protein was extracted from brains of *mpp9*$^{+/+}$ and *mpp9*$^{–/–}$ mice at 1 month after birth and subjected to immunoblotting. GAPDH was used as a loading control. **k** Immunoblots of MPP9 and CEP97 in the kidneys of *mpp9*$^{+/+}$ or *mpp9*$^{–/–}$ mice at 4 months after birth. GAPDH was used as a loading control. **l** Immunostaining of acetylated-tubulin (Acet-Tub, red) in kidneys from the *mpp9*$^{+/+}$ (left) or *mpp9*$^{–/–}$ (right) mice at 4 months after birth. DNA was stained with DAPI (blue). White dashed lines indicate the border of each tubule. **m** Quantification of the percentage of cells with cilia in each tubule in **l**. (*n*= 3 mice for each group). **n** Immunoblotting of MPP9 in *mpp9* $^{+/+}$ and *mpp9* $^{–/–}$ MEF cells. Tubulin was used as a loading control. **o** Immunostaining of MPP9 and γ-tubulin in *mpp9*$^{+/+}$ and *mpp9*$^{–/–}$ MEF cells. **p** Quantifications of the percentage of ciliated cells in *mpp9* $^{+/+}$ and *mpp9* $^{–/–}$ MEF cells. For **b**, **c**, **e**, **f**, **h**, **i**, **m**, and **p**, bars represent the means ± S.E.M for three independent experiments. n.s., not significant; **p,* < 0.05; ***p,* < 0.01; ***, *p* < 0.001; as determined by one-way ANOVA analysis (**b**, **c**, **e**, **f,** and **h**), and unpaired two-tailed Student's *t*-test (**i**, **m**, and **p**). Scale bars: 1 μm (**a, o**); 5 μm (**l**).

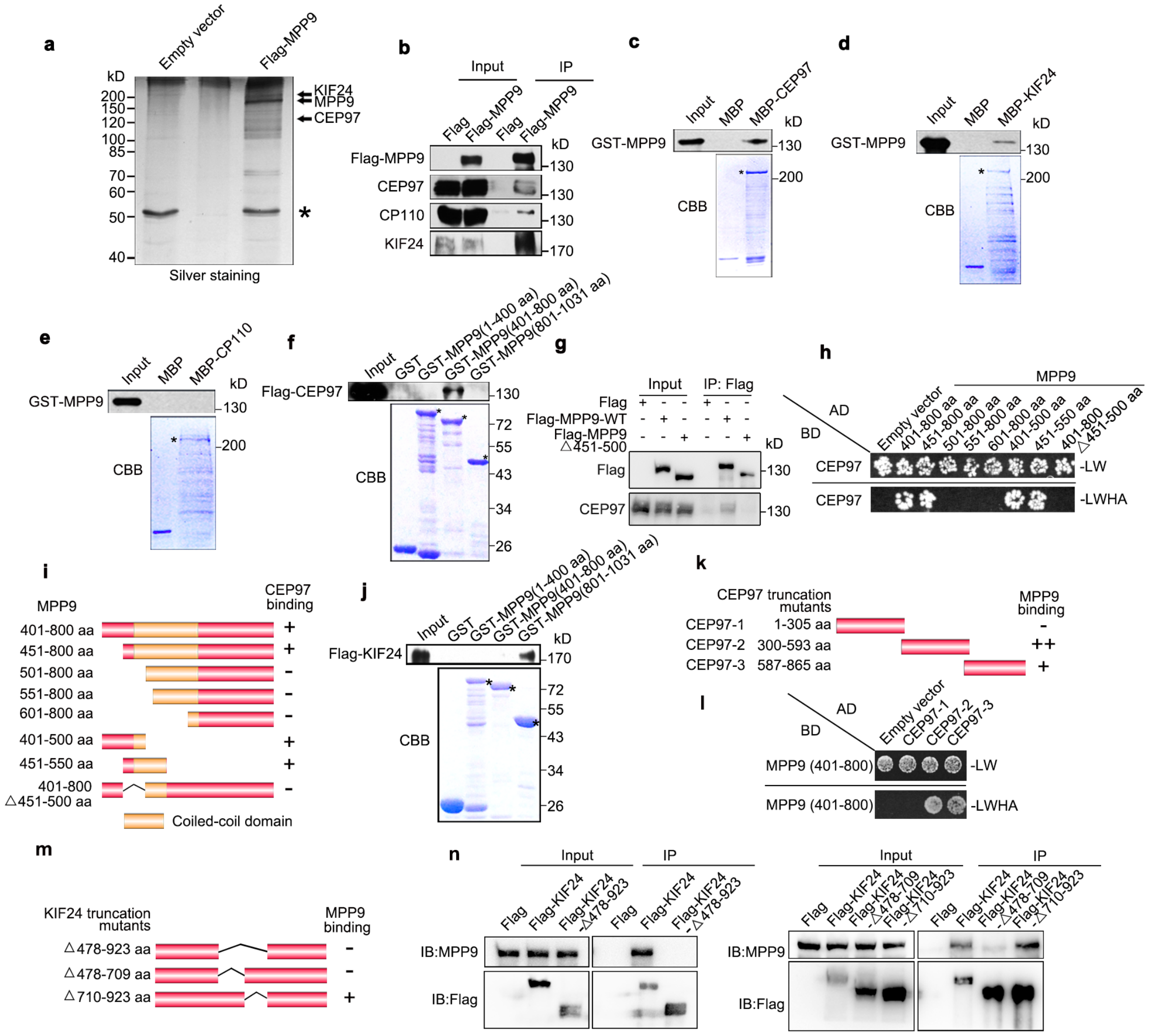

Supplementary Figure 3. MPP9 interacts with KIF24, CEP97, and CP110. a Flag-MPP9 complexes immuno-purified from HEK293T cells were silver stained and subjected to mass spectrometric analysis. Arrows indicate the bands containing MPP9, KIF24, and CEP97. The asterisk marks IgG bands. For screening MPP9 interacting proteins, Flag-MPP9 and Flag-tag empty vector were respectively transfected into HEK293T cells for 48 hours. After immunoprecipitation with anti-Flag antibody, the distinct bands only appeared in the Flag-MPP9 group were subjected to the mass spectrometric analysis. **b** Lysates of HEK293T cells overexpressing Flag-MPP9 were subjected to immunoprecipitation (IP) and immunoblotted with anti-Flag, anti-CEP97, anti-CP110, and anti-KIF24 antibodies. **c-e** *In vitro* binding assays. Purified GST-tagged MPP9 was incubated with amylose resin beads coated with MBP, MBP-CEP97 (**c**), MBP-KIF24 (**d**), or MBP-CP110 (**e**), and immunoblotted with an anti-GST antibody. The asterisks mark the main bands of the recombinant proteins. **f** GST pull-down assays between MPP9 and CEP97. Lysates from HEK293T cells overexpressing Flag-CEP97 were incubated with glutathione-agarose beads coated with GST, GST-MPP9 (1-400 aa), GST-MPP9 (401-800 aa), or GST-MPP9 (801-1031 aa), and immunoblotted with an anti-Flag antibody. The asterisks mark the main bands of the recombinant proteins. **g** Lysates from HEK293T cells overexpressing Flag-tagged MPP9 wild-type (Flag-MPP9-WT) or mutant lacking 451-500 aa (Flag-MPP9-Δ451-500) were subjected to immunoprecipitation (IP) and immunoblotting with anti-Flag and anti-CEP97 antibodies. **h** Yeast two-hybrid assays of CEP97 with the indicated truncates of MPP9.

Transfected yeast cells were plated onto DDO media (-LW) and QDO media (-LWHA). AD, activation domain; BD, binding domain; DDO, double drop out; QDO, quadruple drop out; LW, leucine, tryptophan; LWHA, leucine, tryptophan, histidine, and adenine. **i** Schematic of truncated MPP9 (401-800 aa). CEP97 binding activity: +, positive; –, negative. **j** GST pull-down assays between MPP9 and KIF24. Lysates of HEK293T cells overexpressing Flag-KIF24 were incubated with glutathione-agarose beads coated with GST, GST-MPP9 (1-400 aa), GST-MPP9 (401-800 aa), or GST-MPP9 (801-1031 aa) and immunoblotted with anti-Flag antibody. The asterisks mark the main bands of the recombinant proteins. **k** Schematic of truncates of CEP97. MPP9 binding activity: +, positive; –, negative. **l** Yeast two-hybrid assays of MPP9 (401-800 aa) with truncates of CEP97 from **k**. Transfected yeast cells were plated onto DDO media (-LW) and QDO media (-LWHA). **m** Schematic of truncates of KIF24. MPP9 binding activity: +, positive; –, negative. **n** Lysates from HEK293T cells overexpressing Flag-tagged KIF24 and the indicated truncated mutants were subjected to immunoprecipitation (IP) and immunoblotting (IB) with anti-Flag and anti-MPP9 antibodies.

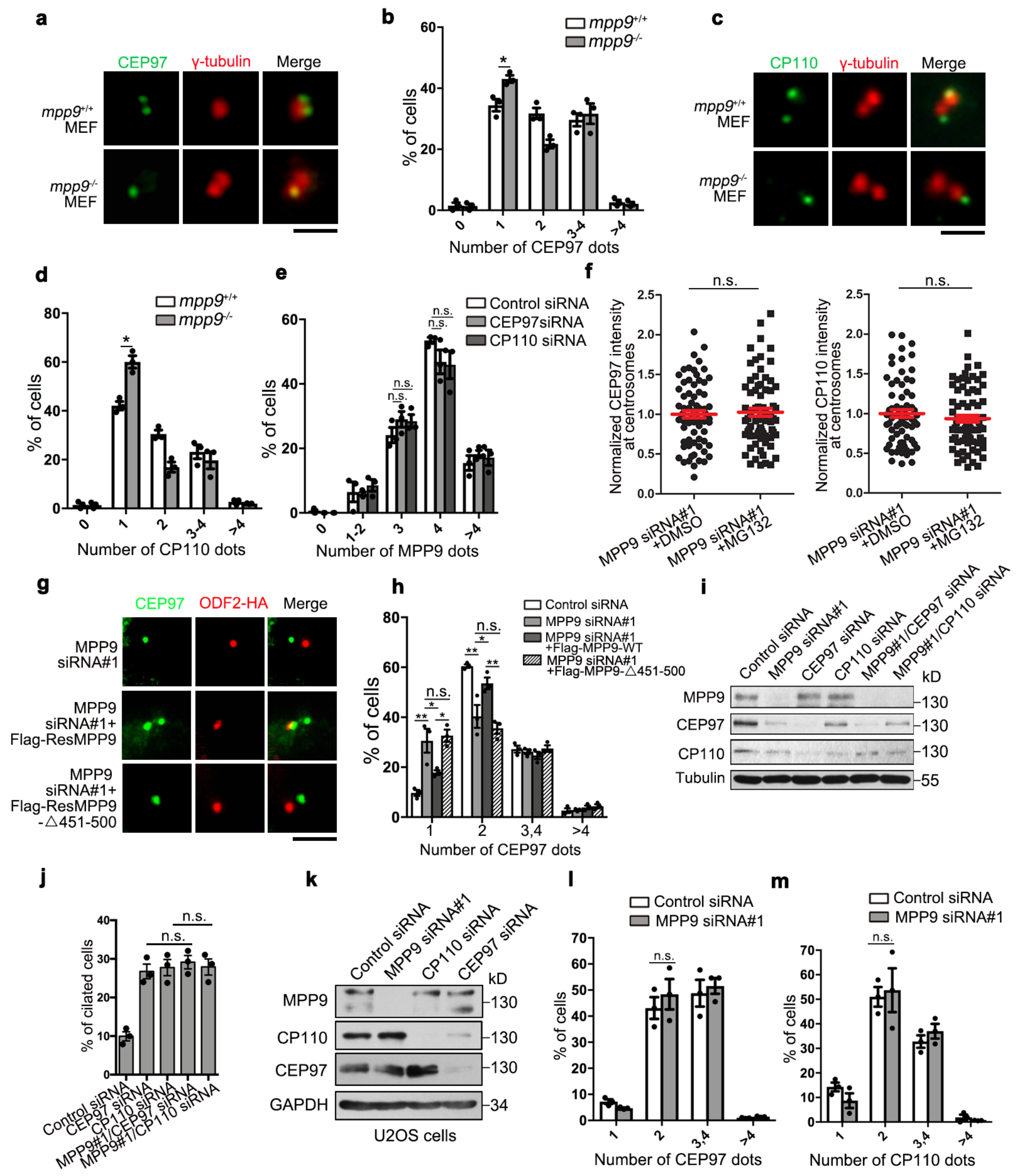

Supplementary Figure 4. MPP9 recruits CEP97 and CP110 to the distal end of the mother centriole in MEF cells, but not in U2OS cells. a Immunostaining of CEP97 (green) and γ-tubulin (red) in the $mpp9^{+/+}$ and $mpp9^{-/-}$ MEF cells. **b** Quantification of the percentage of cells with the indicated number of CEP97 dots at the centrosome from **a**. **c** Immunostaining of CP110 (green) and γ-tubulin (red) in the $mpp9^{+/+}$ and $mpp9^{-/-}$ MEF cells. **d** Quantification of the percentage of cells with the indicated number of CP110 dots from **c**. **e** Quantification of the percentage of cells with the indicated number of MPP9 dots in CEP97- or CP110-depleted hTERT RPE-1 cells. **f** Quantification of CEP97 (left) or CP110 (right) intensity at centrosomes in MPP9-depleted hTERT RPE-1 cells treated with DMSO or MG132 for 4 hours (n= 70 cells for each group). **g** Immunostaining of ODF2-HA (red) and CEP97 (green) after transfection of Flag-siRNA-resistant MPP9 (Flag-ResMPP9) or Flag-ResMPP9-Δ451-500 in MPP9-depleted hTERT RPE-1 cells. **h** Quantification of the percentage of cells with the indicated number of CEP97 dots in MPP9-depleted hTERT RPE-1 cells rescued by Flag-siRNA-resistant MPP9 (Flag-ResMPP9) or Flag-ResMPP9-Δ451-500. **i** Immunoblots of lysates from the indicated siRNAs treated hTERT RPE-1 cells.

Tubulin was used as a loading control. **j** Quantification of ciliogenesis in hTERT RPE-1 cells after transfection of the indicated siRNAs. **k** Immunoblots of lysates from MPP9-, CEP97-, or CP110-siRNA treated U2OS cells. GAPDH was used as a loading control. **l** Quantification of the percentage of cells with the indicated number of CEP97 dots in MPP9-siRNA treated U2OS cells. **m** Quantification of the percentage of cells with the indicated number of CP110 dots in MPP9-siRNA treated U2OS cells. For **b**, **d**, **e**, **f**, **h**, **j**, **l**, and **m**, bars represent the means ± S.E.M for three independent experiments. n.s., not significant; *, $p < 0.05$; **, $p < 0.01$; as determined by unpaired two-tailed Student's *t*-test (**b**, **d**, **f**, **l**, and **m**), and one-way ANOVA (**e**, **h**, **j**). Scale bars: 1 μm (**a**, **c**, and **g**).

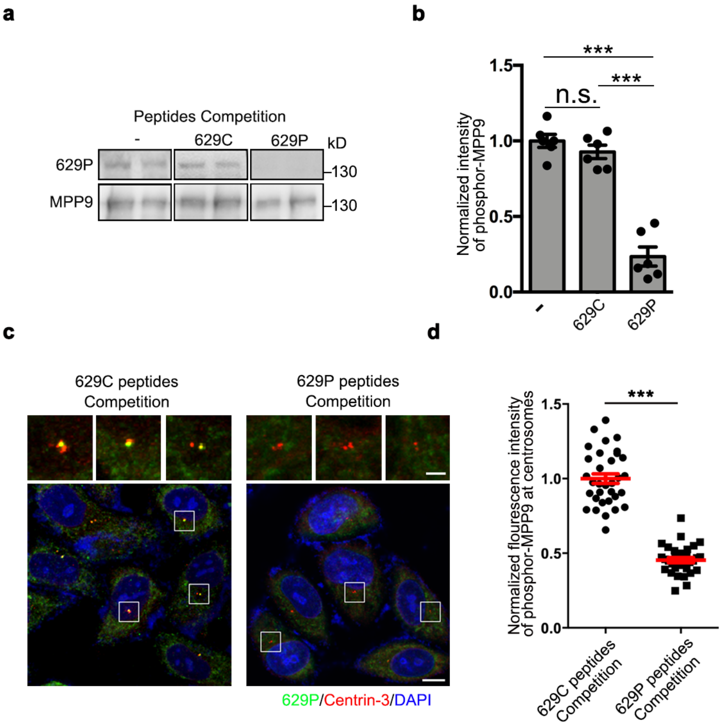

Supplementary Figure 5. The specificity of anti-phospho-MPP9 antibody. a Lysates of hTERT RPE-1 cells after serum starvation and MG132 treatment were subjected to immunoprecipitation (IP) and immunoblotting with anti-MPP9 or anti-phospho-MPP9 antibodies. The antibodies were pre-incubated with the indicated peptides. "–" indicates no peptide pre-incubated group. **b** Quantifications of the intensity of phospho-MPP9 bands from **a**. The intensity of phospho-MPP9 bands was quantified from three repeats, calibrated with total MPP9 levels, and then normalized to no peptide pre-incubated group. **c** Immunostaining of phospho-MPP9 (green) and Centrin-3 (red) in serum-starved hTERT RPE-1 cells. DNA was stained with DAPI (blue). The antibodies were pre-incubated with the indicated peptides. **d** Quantifications of phospho-MPP9 intensity at centrosomes from **c** (n= 33 cells for each group). For **b** and **d**, bars represent the means ± S.E.M for three independent experiments. n.s., not significant; ***, $p < 0.001$; as determined by one-way ANOVA (**b**), and unpaired two-tailed Student's t-test (**d**). Scale bars: 1 µm (**c**, insets); 5 µm (**c**, main).

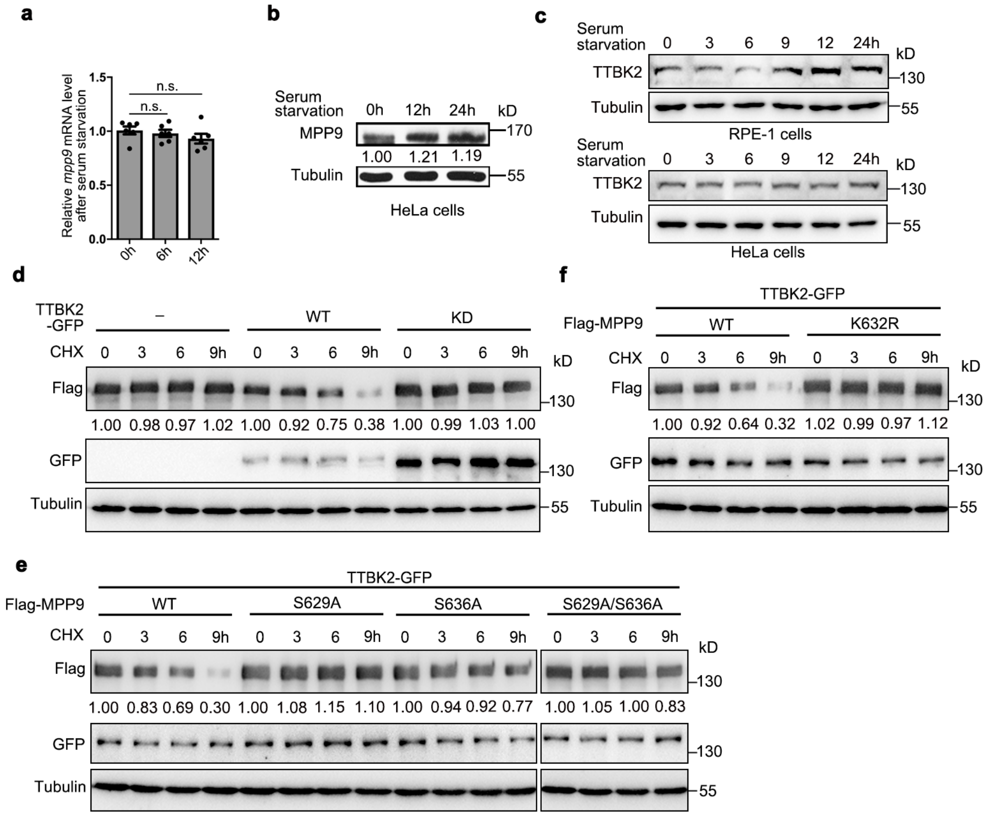

Supplementary Figure 6. TTBK2 accelerates MPP9 degradation. **a** Quantification of *mpp9* mRNA levels after serum starvation in hTERT RPE-1 cells. **b** Immunoblots showing the levels of MPP9 after serum starvation in HeLa cells. Tubulin was used as a loading control. Relative amounts of MPP9 were quantified and normalized to tubulin. **c** Immunoblots of TTBK2 after serum starvation in hTERT RPE-1 cells (upper) or HeLa cells (lower). Tubulin was used as a loading control. **d** Immunoblot analysis of cell lysates derived from Flag-MPP9 stably expressed hTERT RPE-1 cell line transfected with wild-type (WT) or kinase-dead (KD) TTBK2-GFP. Cells were treated with 100 µg ml^{-1} cycloheximide (CHX) before harvesting. Tubulin was used as a loading control. Relative amounts of Flag-MPP9 were quantified and normalized to tubulin. **e** Immunoblot analysis of cell lysates derived from Flag-MPP9 or its non-phosphorylatable mutants (629A, 636A, and 629A/636A) stably expressed hTERT RPE-1 cell lines transfected with TTBK2-GFP. Cells were treated with 100 µg ml^{-1} CHX before harvesting. Tubulin was used as a loading control. Relative amounts of Flag-MPP9 were quantified and normalized to tubulin. **f** Immunoblot analysis of cell lysates derived from Flag-MPP9 wild-type or its non-ubiquitinatable mutant (K632R) stably overexpressed hTERT RPE-1 cell line transfected with TTBK2-GFP. Cells were treated with 100 µg ml^{-1} CHX before harvesting. Tubulin was used as a loading control. Relative amounts of Flag-MPP9 were quantified and normalized to tubulin. For **a**, bars represent the means ± S.E.M for three independent experiments. n.s., not significant, as determined by one-way ANOVA.

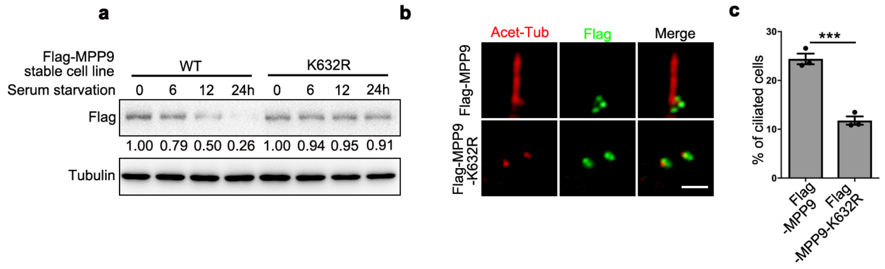

Supplementary Figure 7. Non-ubiquitinatable mutant of MPP9 inhibits ciliogenesis. a Immunoblots of lysates from hTERT RPE-1 cell lines stably overexpressing Flag-tagged MPP9 wild-type (WT) or non-ubiquitinatable mutant (K632R), respectively, after serum starvation. Tubulin was used as a loading control. Relative amounts of Flag-MPP9 were quantified and normalized to tubulin. **b** Immunostaining of acetylated-tubulin (Acet-Tub, red) and Flag (green) in hTERT RPE-1 cell lines stably overexpressing Flag-tagged MPP9 or non-ubiquitinatable mutant (K632R). **c** Quantification of the percentage of cells with cilia from **b**. For **c**, bars represent the means ± S.E.M for three independent experiments. ***, $p < 0.001$, as determined by unpaired two-tailed Student's *t*-test. Scale bar: 1 μm (**b**).

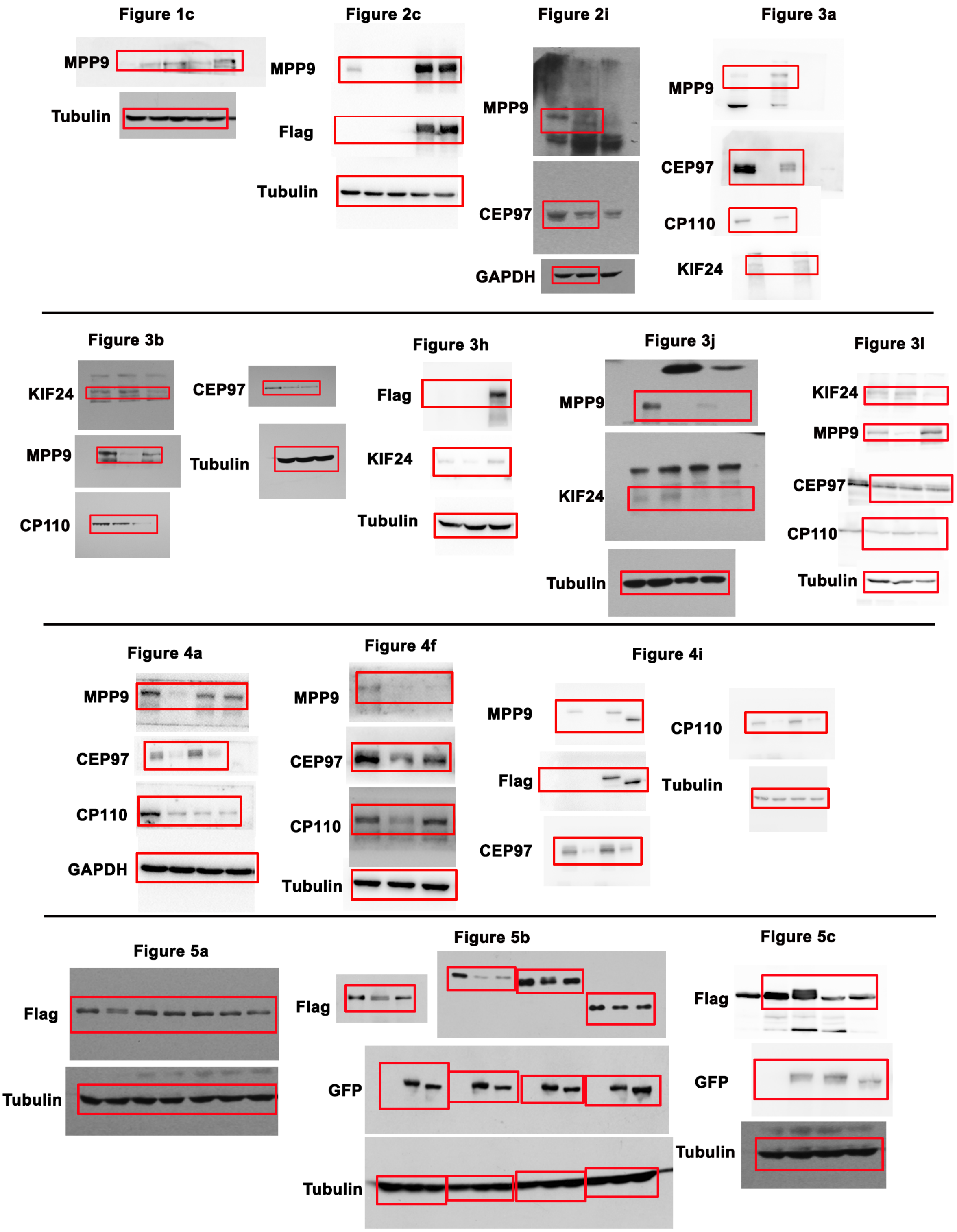
Figure 1c
MPP9
Tubulin
Figure 2c
MPP9
Flag
Tubulin
Figure 2i
MPP9
CEP97
GAPDH
Figure 3a
MPP9
CEP97
CP110
KIF24
Figure 3b
KIF24
CEP97
MPP9
Tubulin
CP110
Figure 3h
Flag
KIF24
Tubulin
Figure 3j
MPP9
KIF24
Tubulin
Figure 3l
KIF24
MPP9
CEP97
CP110
Tubulin
Figure 4a
MPP9
CEP97
CP110
GAPDH
Figure 4f
MPP9
CEP97
CP110
Tubulin
Figure 4i
MPP9
Flag
CEP97
CP110
Tubulin
Figure 5a
Flag
Tubulin
Figure 5b
Flag
GFP
Tubulin
Figure 5c
Flag
GFP
Tubulin

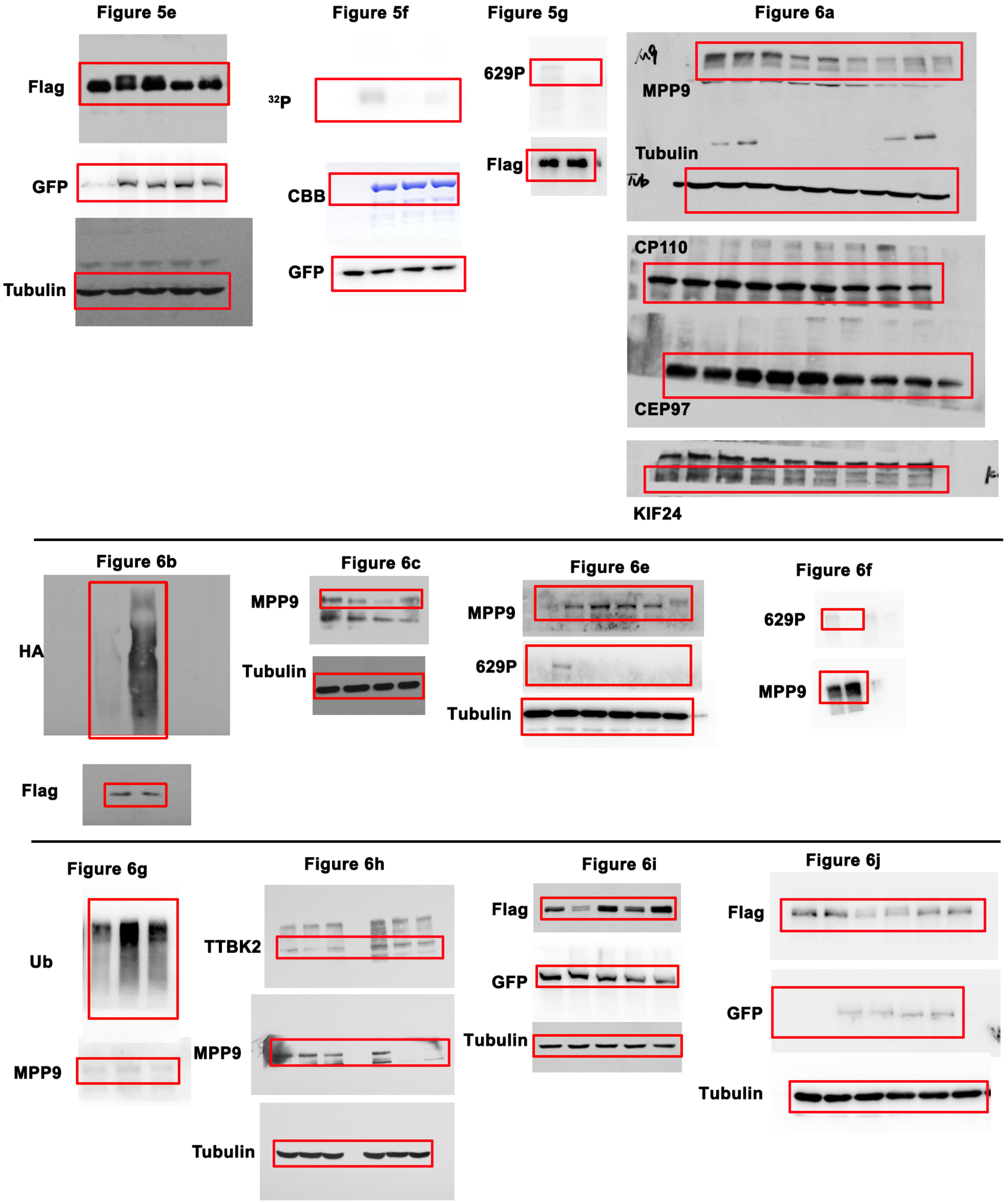
Figure 5e
Flag
GFP
Tubulin
Figure 5f
³²P
CBB
GFP
Figure 5g
629P
Flag
Figure 6a
MPP9
Tubulin
CP110
CEP97
KIF24
Figure 6b
HA
Flag
Figure 6c
MPP9
Tubulin
Figure 6e
MPP9
629P
Tubulin
Figure 6f
629P
MPP9
Figure 6g
Ub
MPP9
Figure 6h
TTBK2
MPP9
Tubulin
Figure 6i
Flag
GFP
Tubulin
Figure 6j
Flag
GFP
Tubulin

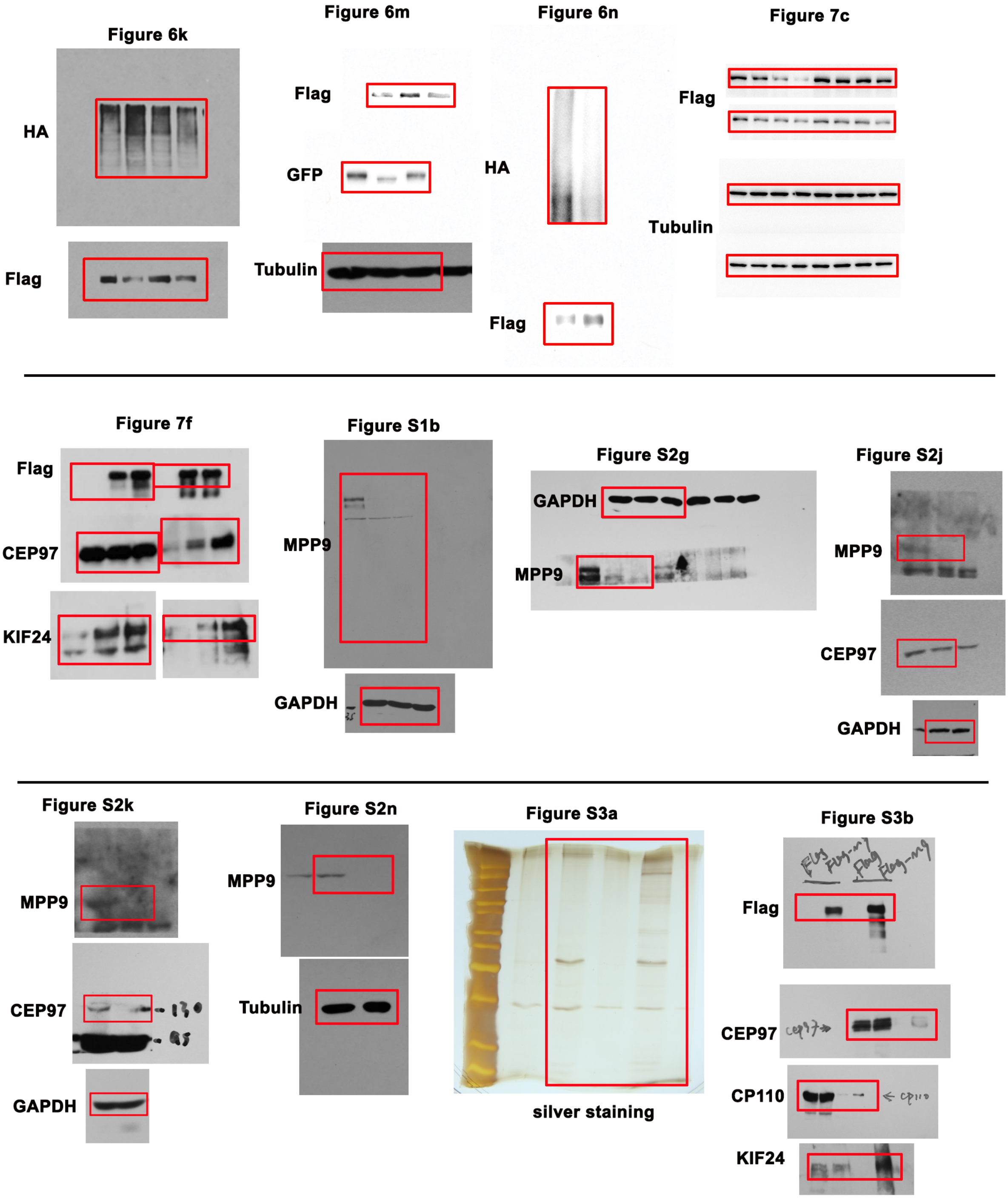
Figure 6k
HA
Flag
Figure 6m
Flag
GFP
Tubulin
Figure 6n
HA
Flag
Figure 7c
Flag
Tubulin
Figure 7f
Flag
CEP97
KIF24
Figure S1b
MPP9
GAPDH
Figure S2g
GAPDH
MPP9
Figure S2j
MPP9
CEP97
GAPDH
Figure S2k
MPP9
CEP97
GAPDH
Figure S2n
MPP9
Tubulin
Figure S3a
silver staining
Figure S3b
Flag
CEP97
CP110
KIF24

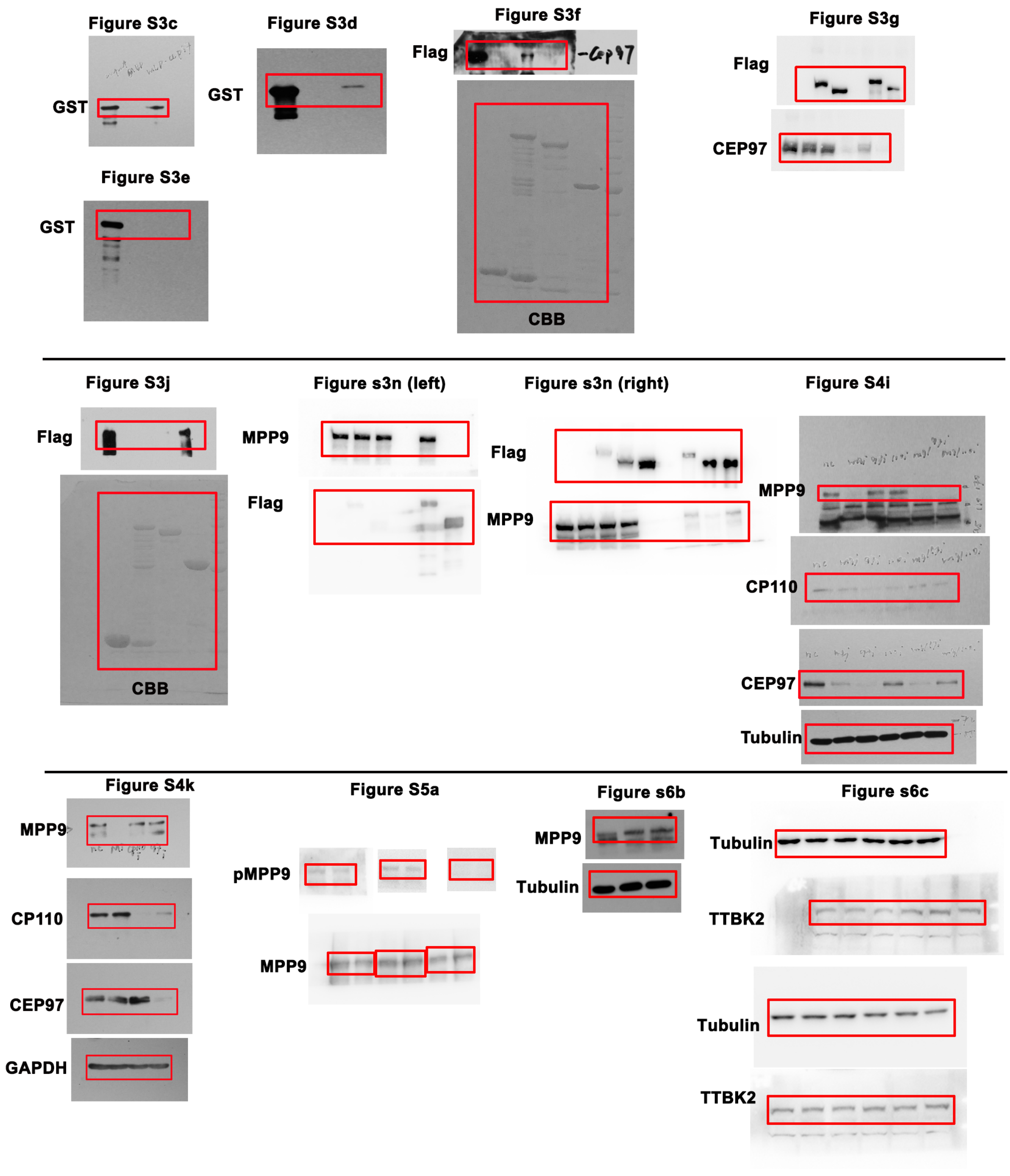
Figure S3c
GST
Figure S3d
GST
Figure S3f
Flag
CBB
Figure S3g
Flag
CEP97
Figure S3e
GST
Figure S3j
Flag
CBB
Figure s3n (left)
MPP9
Flag
Figure s3n (right)
Flag
MPP9
Figure S4i
MPP9
CP110
CEP97
Tubulin
Figure S4k
MPP9
CP110
CEP97
GAPDH
Figure S5a
pMPP9
MPP9
Figure s6b
MPP9
Tubulin
Figure s6c
Tubulin
TTBK2
Tubulin
TTBK2

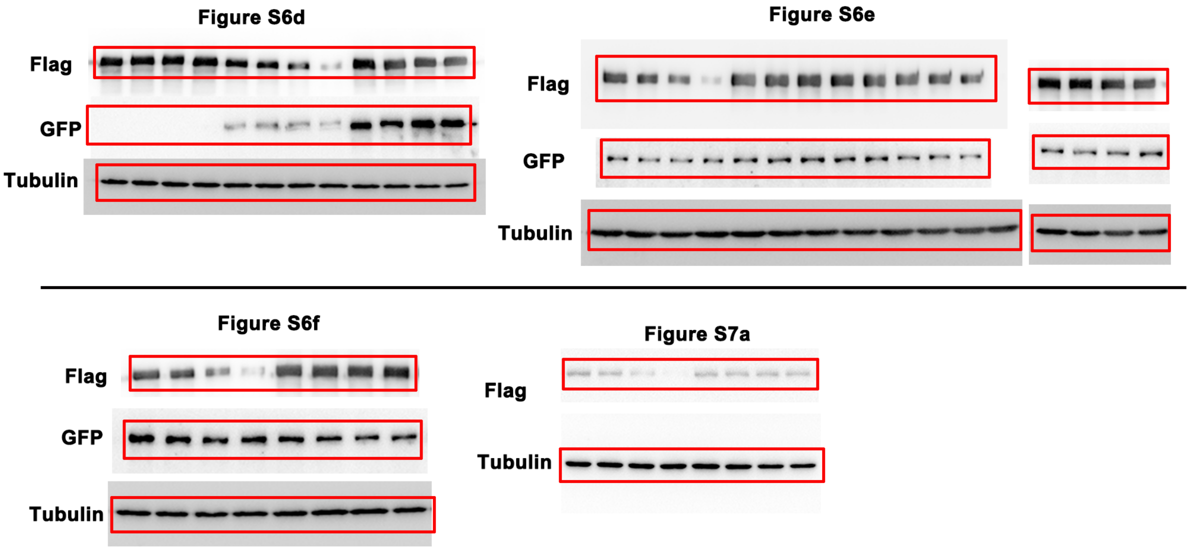

Supplementary Figure 8. Uncropped western blot images of the indicated figures.

Supplementary Table 1. List of antibodies

Names	Source	Identifier	Dilution for IF	Dilution for WB
Rabbit anti-MPP9	Sigma-Aldrich	Cat# HPA037485	1:200	1:1000
Mouse anti-MPP9	This paper	N/A	1:100	1:500
Rabbit anti-CP110	Proteintech	Cat# 12780-1-AP	1:200	1:500
Rabbit anti-CEP97	Proteintech	Cat# 22050-1-AP	1:200	1:1000
Rabbit anti-TTBK2	Sigma-Aldrich	Cat# HPA018113	1:100	1:500
Mouse anti-Centrin-3	Abnova	Cat# H00001070-M01	1:100	N/A
Mouse anti-C-Nap1	Santa Cruz Biotechnology	Cat# sc-390540	1:100	N/A
Rabbit anti-CEP164	Proteintech	Cat# 22227-1-AP	1:100	N/A
Rabbit anti-Arl13b	Proteintech	Cat# 17711-1-AP	1:200	N/A
Rabbit anti-Ubiquitin	Proteintech	Cat# 10201-2-AP	N/A	1:1000
Rabbit anti-GFP	This paper	N/A	N/A	1:2000
Rabbit anti-γ-tubulin	Sigma-Aldrich	Cat# T5192	1:200	N/A
Mouse anti-α-tubulin	Sigma-Aldrich	Cat# T9026	1:500	1:2000
Mouse anti-Flag	Sigma-Aldrich	Cat# F1804	1:200	1:2000
Mouse anti-HA	Sigma-Aldrich	Cat# H9658	N/A	1:2000
Mouse anti-GAPDH	CWBIO	Cat# CW0100	N/A	1:1000
Mouse anti-acetylated tubulin	Sigma-Aldrich	Cat# T7451	1:500	N/A
Rabbit anti-KIF24	This paper	N/A	1:100	1:1000
Rabbit anti-phosphorylated S629-MPP9	This paper	N/A	1:100	1:500
Rabbit anti-Ki67	Sino Biological	Cat# 100130-T32-50	1:100	N/A
Anti-rabbit IgG-Gold (10 nm, colloidal gold)	Sigma-Aldrich	Cat# G7402	1:10	N/A
Anti-Rabbit IgG (H+L)	Jackson ImmunoResearch	Cat# 111-035-003	N/A	1:5000
Anti-Mouse IgG (H+L)	Jackson ImmunoResearch	Cat# 115-035-003	N/A	1:5000
Goat anti-Rabbit IgG (H+L) Highly Cross-Adsorbed Secondary Antibody, Alexa Fluor 488	Invitrogen	Cat# A-11034	1:200	N/A
Goat anti-Mouse IgG (H+L) Highly Cross-Adsorbed Secondary Antibody, Alexa Fluor 488	Invitrogen	Cat# A-11029	1:200	N/A
Goat anti-Rabbit IgG (H+L) Highly Cross-Adsorbed Secondary Antibody, Alexa Fluor 568	Invitrogen	Cat# A-11036	1:200	N/A
Goat anti-Mouse IgG (H+L) Highly Cross-Adsorbed Secondary Antibody, Alexa Fluor 568	Invitrogen	Cat# A-11031	1:200	N/A
Goat anti-Mouse IgG (H+L) Highly Cross-Adsorbed Secondary Antibody, Alexa Fluor 647	Invitrogen	Cat# A-32728	1:200	N/A

Goat anti-Rabbit IgG (H+L) Highly Cross-Adsorbed Secondary Antibody, Alexa Fluor 647	Invitrogen	Cat# A21245	1:200	N/A

Supplementary Table 2. List of siRNA sequences

Names	Sequences (from 5' to 3')
Human siRNA targeting sequence: MPP9 #1	CGCUAAAGAAAUUCCAUGUUTT
Human siRNA targeting sequence: MPP9 #2	GCCACCGAUAACCAUGUUAATT
Mouse siRNA targeting sequence: MPP9 #1	GGAAAGCAAAUAGUGGUAATT
Mouse siRNA targeting sequence: MPP9 #2	GGAGAUAAGUAGUCUGAAATT
Mouse siRNA targeting sequence: MPP9 #3	GGGCGAGUAAAGGAUACAUTT
Human siRNA targeting sequence: CP110	GCAGCAUGAGUAUGCCAGUTT
Human siRNA targeting sequence: CEP97	GAUGAGAAGUGAAAUCAAUTT
Human siRNA targeting sequence: KIF24#1	GGAAGAAAGCUCCGAAAUATT
Human siRNA targeting sequence: KIF24#2	GGAACACCCUGGAGAAUAGTT
Human siRNA targeting sequence TTBK2	CCAUAUCUCUUCUUUGGAUTT
Negative control siRNA	UUCUCCGAACGUGUGUCACGUTT
